# Biopolymer Optical Fibers for High-Sensitivity Quantitative Humidity Monitoring

**DOI:** 10.1021/acsami.5c10056

**Published:** 2025-08-25

**Authors:** Jani Patrakka, Ville Hynninen, Petteri Huttunen, Nonappa

**Affiliations:** † Faculty of Engineering and Natural Sciences, 7840 Tampere University, Korkeakoulunkatu 6, Tampere FI-33720, Finland; ‡ Faculty of Built Environment, 7840 Tampere University, Korkeakoulunkatu 5, Tampere FI-33720, Finland

**Keywords:** biopolymers, optical fibers, humidity sensors, quantitative sensing, composites

## Abstract

Because of their tunable refractive index and surface functionalities, biopolymers have emerged as excellent candidates for the fabrication of sustainable optical fibers. To date, the focus has been on identifying suitable biopolymers for optical fiber fabrication and their associated waveguiding properties. Despite a few studies showing their potential for short-distance applications and humidity sensing, the quantitative sensing of environmental parameters using biopolymer optical fibers has not been reported. Herein, for the first time, we report the quantitative determination of relative humidity (RH) across the visible and near-infrared region of the electromagnetic spectra using fully biopolymer optical fibers. Specifically, we demonstrate that methylcellulose and methylcellulose-alginate composite optical fibers exhibit sensitivity of up to 0.33 dB/%RH. Notably, the sensitivity of our fibers is equal to or greater than most of the optical sensors reported in recent literature and exceeds the reported values for capacitance-based humidity sensors. The sensitivity, response time, and dynamic range can be readily tuned by changing the fiber composition and coagulation methods, with ethanol coagulated composite fibers displaying a 6-fold increase in sensitivity compared to ionically coagulated composite fibers. Our results suggest that the humidity sensing properties of fiber sensors are insensitive to temperature changes. Dynamic Vapor Sorption analysis reveals that the fiber composition regulates the sorption–desorption kinetics, thereby affecting the humidity sensing behavior. The fully biopolymer optical fibers open a new avenue for sustainable sensors that enable the quantitative sensing of environmental parameters for condition monitoring applications.

## Graphical abstract

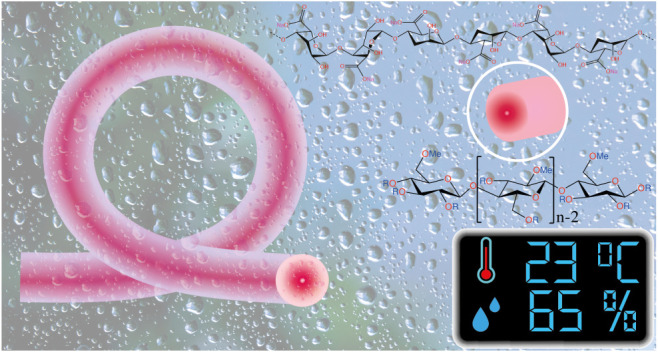

## 1 Introduction

Humidity is one of the vital environmental parameters that affect most processes occurring in ambient conditions.^1^ High humidity can be observed as fog or precipitation, making perspiration-based thermoregulation difficult, while arid conditions can lead to reduced human comfort, such as nasal tissue drying.^2^ In addition to health and comfort, accurate control of humidity and other environmental conditions is vital in agriculture, meteorology, food processing and storage, museums and archive storage, chemical and pharmaceutical storage, and semiconductor manufacturing to ensure the quality and consistency of goods.^3–6^ Relative humidity (RH) is the most common humidity measurement quantity, and it refers to the percentage of water in the air relative to the saturated value.^1^ RH is routinely measured using capacitive or resistive electronic sensors. Modern low-cost capacitive humidity sensors utilize materials such as metal oxides, porous ceramics, silicon, or polymers as the sensing elements.^7^ The sensing material layer with humidity-dependent relative permittivity, *ϵ*_*r*_(*RH*) is sandwiched between electrodes measuring capacitance over the sensing layer and translating the readings into RH readings.^8^ However, capacitive sensors are sensitive to temperature changes, affecting the dielectric constant of humidity-sensitive materials, requiring simultaneous temperature monitoring for accurate humidity readings.^9–11^ Furthermore, water condensation affects the recovery time in high humidity environments, making it challenging to use capacitive sensors in high and rapidly changing conditions. Prolonged exposure to humidity also results in hysteresis. Resistive sensors, on the other hand, exhibit lower accuracy compared to capacitance sensors and require additional linearization techniques to compensate for nonlinear responses to humidity changes.^9^

In recent years, optical fiber-based humidity sensors have gained prominence over electronic sensors.^12,13^ This is attributed to their high sensitivity, the possibility of measuring distributed sensing along the length of the fiber, and their immunity to electromagnetic interferences. Optical fiber-based sensors typically operate by detecting variations in light intensity or wavelength caused by changes in the refractive index (RI) of humidity-sensitive materials. Since its discovery, silica glass optical fibers (GOFs) have evolved as one of the most important candidates for fiber optic-based sensors.^14,15^ GOFs have been utilized as RH sensing platforms using various approaches, including fiber Bragg gratings, modal interferometers, evanescent wave sensors, and Fabry–Pérot interferometers.^16–23^ However, GOFs lack intrinsic humidity sensing properties, requiring coating with humidity sensitive materials. Polymer optical fibers (POFs) are lightweight, exhibit fatigue tolerance, and possess mechanical robustness.^24,25^ However, commercially used POFs made from polystyrene (PS) and poly­(methyl methacrylate) (PMMA) lack intrinsic humidity sensing properties (see Figure S1). Both GOFs and POFs require sophisticated fabrication and coating of humidity sensitive materials such as polyimide (PI), poly­(vinyl alcohol) (PVA), silicon dioxide (SiO_2_), or tin oxide (SnO_2_).^26–29^ Additionally, several biopolymers such as agarose, gelatin, chitosan, and alginate (Alg) have been utilized as humidity sensing coating materials in GOFs and POFs-based sensors.^30–35^ Therefore, the sensor performance and sensitivity depend on the properties of coated sensing materials.

Recently, biocompatible synthetic polymers,^36–39^ and biopolymer optical fibers (BOFs) have emerged as potential candidates for short-distance applications.^40–45^ While a majority of studies focus on the waveguiding properties of BOFs, some reports have highlighted their environmental sensing applications. Due to their biodegradability, flexibility, and compatibility with biological tissues, biopolymer-based waveguides are ideal candidates for wearable photonic devices for continuous, noninvasive monitoring of physiological conditions and activity.^46,47^ For example, Li et al. fabricated a wearable photonic wristband that integrates skin-conformal optical fibers, enabling noninvasive monitoring of cardiorespiratory signals and facilitating biometric identification for health monitoring and personal authentication.^48^ In another study, the same research group demonstrated a highly sensitive, sandwich-structured optical fiber for detecting subtle throat vibrations in bilingual speech recognition and silent communication, a tool for assistive voice technologies and encrypted communication.^49^ Biopolymers such as natural and genetically engineered silk, deoxyribonucleic acid (DNA), chitosan, agarose, alginates, and regenerated cellulose have been studied for their waveguiding properties with varying degrees of success.^40–45^ Silk-based optical waveguides exhibit a moderate response to humidity. However, highly sophisticated techniques are required for the preparation of genetically engineered silk, which is both cost-intensive and has limited scalability and surface modification capabilities. Hydrogel optical fibers fabricated using agarose and alginate also display strong humidity sensitivity. However, hydrogel fibers face challenges due to dehydration and swelling, limiting their application in extreme humidity conditions. Furthermore, above a certain solid content, the hydrogels become brittle and challenging to handle. Among biopolymers, cellulose is naturally abundant, renewable, and allows facile chemical modification. Cellulose and its derivatives offer tunable refractive index (1.40–1.59) and nearly 90% transmission above 300 nm in the electromagnetic spectrum.^50,51^ Furthermore, they form shear-thinning hydrogels, allowing ready extrusion of fibers under ambient conditions.^52,53^ Cellulose-based fibers are readily scalable, display superior mechanical strength, and maintain their structures over a wide range of environmental conditions compared to other biopolymer-based fibers.^52^ More importantly, cellulose naturally exhibits strong absorption and desorption of water, making it suitable for rapid humidity sensing,^54^ and integration into smart textiles.^55^ Dupuis et al. reported cellulose butyrate and hydroxypropyl cellulose-based optical fibers with a transmission loss of between 1 and 2 dB cm^–1^ at 630 nm.^56^ However, the fibers showed sample-to-sample variation in light transmission due to nonuniform structure. Orelma et al. utilized an ionic liquid for the fabrication of regenerated cellulose-cellulose acetate core-cladding fibers, showing an optical loss of 6.3 dB cm^–1^ at 1300 nm.^57^ In another report, Reimer et al. demonstrated a minimum transmission loss of 0.56–0.82 dB cm^–1^ at 860 nm for regenerated cellulose-based optical fiber.^58^ We have previously reported the methylcellulose (MC)-based luminescent optical fiber with anattenuation coefficient of 1.47 dB cm^–1^.^59,60^ In another study, we demonstrated that carboxymethyl cellulose-based optical fibers enable short-distance communication and environmental sensing.^61^ More recently, we have demonstrated methylcellulose-alginate (MC-Alg) composite optical fibers with enhanced performance in the near-infrared (NIR) region.^62^ Even though sensing applications have been demonstrated, so far, no quantitative humidity sensing has been demonstrated using a fully biobased optical fiber sensor. Here, we report on the quantitative humidity sensing of biopolymer optical fibers for the first time. Specifically, we show that one-component BOF fabricated using methylcellulose or alginate allows quantitative humidity sensing with sensitivity ranging from 0.05 dB/%RH to 0.152 dB/%RH. Notably, in composite MC-Alg BOFs, the sensitivity was doubled to 0.33 dB/%RH. By comparing more than 100 literature reports, we show that the sensitivity of BOFs is equal to or greater than that of optical sensors and exceeds that of capacitance-based RH sensors. Furthermore, the coagulation conditions affected the sensitivity, with ethanol coagulated composite BOFs showing a 6-fold increase in sensitivity compared to ionically coagulated composite BOFs. Using variable-temperature measurements of pristine and preheated fibers, we demonstrate that humidity sensing is independent of temperature. The Dynamic Vapor Sorption (DVS) analysis further supports that the water sorption–desorption is regulated by the fiber composition and coagulation conditions.

## 2 Experimental Section

### 2.1 Materials and Methods

Methylcellulose, MC (molecular weight 88 000 g/mol, product no. M0512), sodium alginate (alginic acid sodium salt), and calcium chloride (CaCl_2_, ≥96%) were purchased from Sigma-Aldrich and used as received. Absolute ethanol (99.7 vol/vol % Etax Aa, Altia Inc.) was used in the coagulation bath (diluted with water to obtain 96:4 ethanol:water v/v) during fiber spinning. Ultrapure Milli-Q water (18.2 MΩ cm) was used to prepare the spinning dope and coagulation solution in all experiments.

### 2.2 Preparation of Alginate Spinning Dope

To prepare a 4% spinning dope, 4.0 g of sodium alginate powder was added portion-wise to a glass bottle containing 100 mL of ultrapure Milli-Q water, stirred (∼1000 rpm) at room temperature (22–23 °C). The mixture was stirred until a clear dispersion was obtained. The spinning dope was stored at +4 °C until further use.

### 2.3 Preparation of Methylcellulose Spinning Dope

To prepare a 4% spinning dope, 4.0 g of methylcellulose was added portion-wise to a glass bottle containing 100 mL of ultrapure Milli-Q water, stirred (∼1000 rpm) at 85 °C to obtain a homogeneous dispersion. The resulting cloudy dispersion was cooled using an ice bath to obtain a transparent spinning dope. During cooling, a shaking was applied to prevent any phase separation or sedimentation. The spinning dope was stored at +4 °C until further use.

### 2.4 Preparation of Methylcellulose-Alginate Composite Spinning Dope

To prepare a spinning dope containing a 3:1 ratio of methylcellulose and alginate (MC-Alg), 3.0 g of MC and 1.0 g of Alg were mixed and added portion-wise to a glass bottle containing 100 mL of ultrapure Milli-Q water under constant stirring (∼1000 rpm) at room temperature (22–23 °C) to obtain a clear solution.

### 2.5 Preparation of CaCl_2_ Solution

The calcium chloride (5%) was prepared by adding 10.0 g of CaCl_2_ portion-wise to a glass bottle containing 200 mL of ultrapure water under constant stirring (∼1000 rpm) at room temperature (22–23 °C) to obtain a clear solution. The solution was used as a coagulation bath for the Alg and MC-Alg composite fiber fabrication.

### 2.6 Fiber Fabrication and Characterization

Fiber fabrication was performed using a wet-spinning method following our previously reported procedure (Figure 1a).^62^ Briefly, in a typical experiment, a spinning dope loaded stainless-steel syringe (6 or 20 mL) was attached to the Fusion 6000 Syringe Pump (Chemyx), fitted with a 1.0 m long polyether ether ketone (PEEK) extrusion tube (diameter, Ø = 0.727 mm). The fiber extrusion was performed with a flow rate of 1.8 mL/min into a coagulation bath. The ethanol coagulated fibers were equilibrated for 20 min in the coagulation bath. Ionically coagulated fibers were stabilized for 3 min in the coagulation bath, followed by rinsing with ultrapure Milli Q water for 2 min to remove excess ions. Thereafter, the fibers were dried under ambient conditions by suspending them vertically between the glass rods. The scanning electron microscopy (SEM) imaging was performed by attaching the dry fibers to a carbon tape placed on an aluminum stub, followed by sputter coating with platinum–palladium (6 nm) using a Leica EM ACE600 high-vacuum sputter coater. The imaging was performed using Zeiss Ultra FESEM operating at an acceleration voltage of 5 kV.

**Figure 1:**
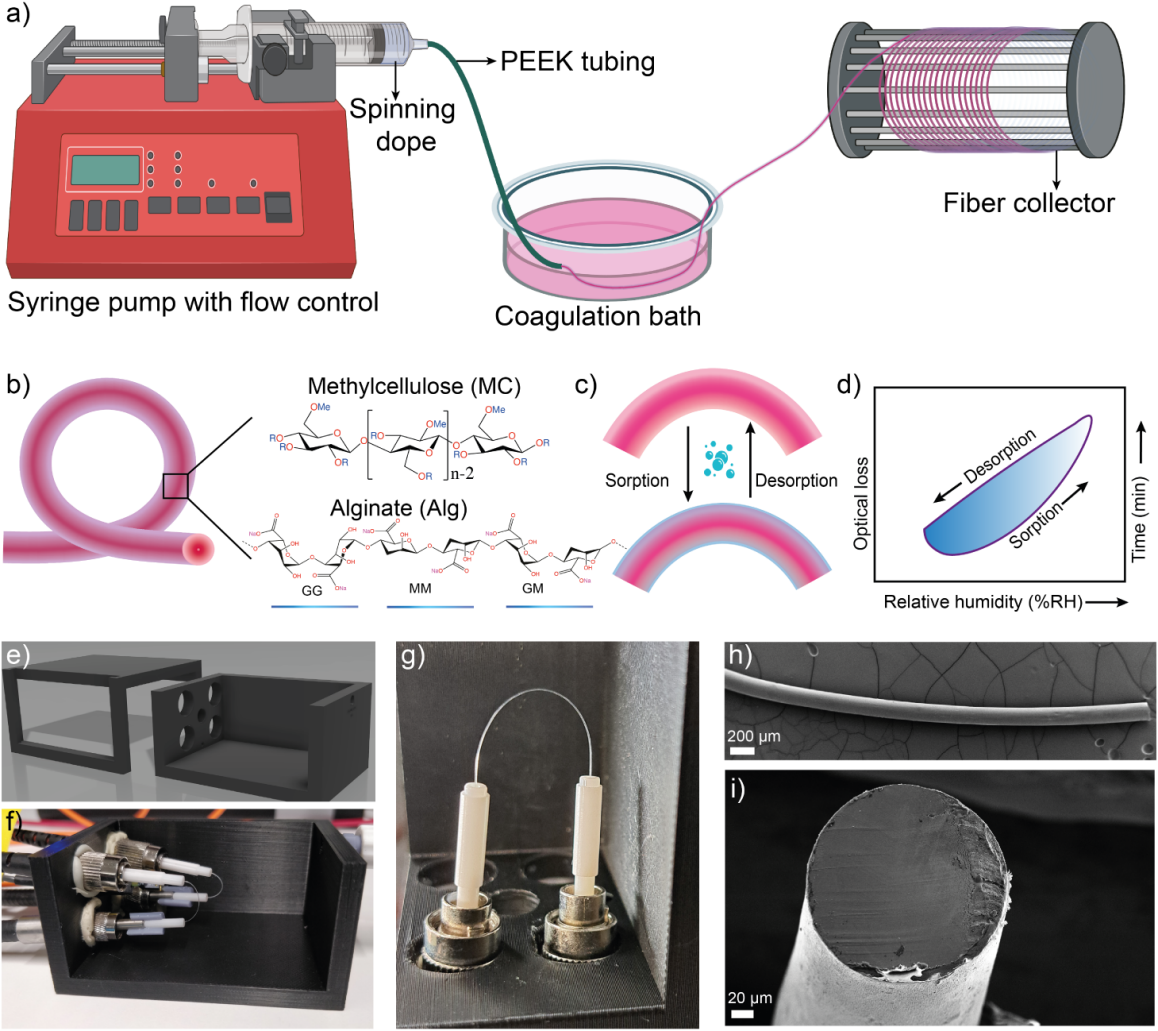
Biopolymer optical fiber fabrication and humidity sensitivity. (a) Schematic illustration of the wet-spinning process. (b) Schematic representation of biopolymer optical fiber and chemical structures of MC, and Alg. (c) Biopolymers allow dynamic vapor sorption, and as a result, dynamic cladding is formed. (d) Dynamic sorption–desorption results in humidity dependent transmission changes, allowing quantitative determination of RH. (e) 3D model of a custom-made humidity chamber. (f) A photograph of biopolymer optical fibers coupled into the custom humidity chamber. (g) Photograph of a BOF inside the humidity chamber used for sensing. (h) SEM image of a composite MC-Alg BOF. (i) SEM image showing the cross-sectional view of an MC-Alg BOF. (a) was created using Biorender.com.

### 2.7 Near-Infrared (NIR) Attenuation Measurements

The attenuation coefficient was estimated using the cutback method, as previously reported (see Supporting Information for details).^62^ In brief, an NIR spectrometer was used to record transmitted white light from a halogen lamp (Ocean Optics DH-2000-BAL, 230 – 2500 nm), and the Beer–Lambert law was used to determine attenuation from optical transmission at varying fiber length. The errors in attenuation were calculated to be at most 3.3% using the propagation of uncertainty.

### 2.8 Humidity Sensitivity of Fibers

Optical response to humidity changes in fibers was recorded across UV–Vis and NIR wavelength ranges using Avantes AvaSpec ULS2048L and AvaSpec-NIR-1.7 spectrometers, respectively. Transmission spectra were determined from photon count spectra between 300 and 1100 at 0.6 nm spectral resolution and 900 and 1700 at 4 nm spectral resolution. The transmissions spectra for each fiber type were measured from 5 sample pieces, and 3–5 of the measure spectra were used to determine the representative average transmission spectrum of each sample type. White light from deuterium and halogen lamps (Ocean Optics DH-2000-BAL, 230–2500 nm) was guided through a commercial multimode fiber (Thorlabs M12L01, Ø300 μm, 0.39 NA) into the samples and subsequently collected with a similar commercial multimode fiber from the samples into the spectrometer. The spectrometer integration time was adjusted to ensure a high photon count, and the resulting spectra were compared to the integration-time-adjusted baseline from the setup without a sample. For the measurement, fiber samples were prepared by cutting them into a suitable length (3 ± 0.1 cm), and the tips were then butt-coupled into the commercial multimode fibers for light input and output. Custom made 3D printed ferrule sleeves were used to couple the commercial fibers with the experimental fiber. Vis and NIR spectra are presented in decibels (dB) to keep the results comparable with the literature (conversion algorithm explained in more detail in Supporting Information S2), and the formula for conversion from photon counts to logarithmic optical losses is

1 $$\mathrm{Attenuation}\left(\lambda \right)\;[\mathrm{dB}]=-10\cdot \log_{10}\frac{I_{s}\left(\lambda \right)}{I_{B}\left(\lambda \right)\cdot \Delta t}$$

where the optical losses or attenuation at wavelength λ is determined by the ratio of photon counts from the sample *I_s_* to the baseline photon counts from the setup without a sample *I_B_*. In the case that the sample and baseline measurements differ by integration time, the baseline photon count is multiplied by the ratio of sample and baseline integration times $\Delta t=\frac{t_{s}}{t_{B}}$ . The response time for humidity changes was evaluated with repeated cycles of 10–20% RH changes and by comparing the optical signal change to the reference sensor of the humidity controller unit (Figure S3). In our previous work, a subsecond response to RH changes from breathing was observed in a similar 3–5 dB magnitude.^62^

In addition to the length-dependent attenuation, transmission through the BOFs was affected by the coupling losses between commercial multimode fibers and experimental BOFs in the setup. The coupling losses were estimated using the cutback method (see Supporting Information for details) to be 10–20 dB, depending on the type of sample. The estimation was made by comparing the extrapolated transmission through the fiber at 0 cm length to the baseline of the system without BOFs. The relatively soft BOF tips were challenging to polish, resulting in suboptimal fiber cleaving and possible air gaps between the fiber tips. Numerical aperture mismatch and different fiber core sizes also contribute to the mode mismatch and reduced coupling efficiency. Furthermore, a geometrical mismatch was the most likely cause for high coupling losses as the input fiber core (Ø300 μm) was often larger than the experimental fiber core (Ø200–300 μm). Moreover, the ethanol-coagulated BOFs had a flattened, more elliptical cross-section, leading to some input light not being coupled into the BOFs. Optical return losses also play a role in the total coupling losses. Measurements were conducted in a custom 3D printed measurement chamber with humidity-controlled air input and a small hole for airflow output. Commercial multimode fiber tips were secured within the chamber to fully expose the experimental fiber sample to varying humidity. Input air humidity was controlled with a Linkam RH95 Humidity Controller, and airflow was guided into the measurement chamber through Luer-tapered Tygon tubing. The Linkam RH sensor was also fixed within the chamber to monitor the changes in humidity that caused changes in light transmission through the fiber. Spectral data from sample sets were analyzed with MATLAB to produce a mean transmission spectrum representing the sample set. As the white light source has an emission stability of 0.01%/h and the Linkam humidity controller sensor accuracy is 1.5% RH, the errors in humidity sensitivity were calculated with the propagation of uncertainty to be at most 4.0%.

### 2.9 Thermal Sensitivity of Fibers

Optical response to temperature changes in fibers was recorded across the UV–Vis range using an Avantes AvaSpec ULS2048L spectrometer. Transmission spectra were determined from photon count spectra between 300 and 1100 at 0.6 nm spectral resolution. White light from deuterium and halogen lamps (Ocean Optics DH-2000-BAL, 230–2500 nm) was guided through a commercial multimode fiber (Thorlabs M12L01, Ø300 μm, 0.39 NA) into the samples and subsequently collected with a similar commercial multimode fiber from the samples into the spectrometer. A standard heat plate was used for BOF temperature ramping between 20 and 100 °C, and transmitted light was monitored during heating and cooling. As the standard heat plate has a temperature accuracy of ±1 °C, the errors in temperature sensitivity were calculated using the propagation of uncertainty to be at most 3.6%.

### 2.10 Dynamic Vapor Sorption (DVS) Analysis

The hygroscopicity of BOFs was measured using DVS Adventure from Surface Measurement Systems, having a maximum sample weight of 1.5 g and an experimental dynamic range of 150 mg. Fiber samples were cut into 10–15 cm pieces from the stock and bundled into a spherical shape to fit into the sample holder crucible hanging from an ultrabalance hook (0.01 μg resolution with ≤0.3 μg RMS noise). Samples were exposed to cycles with drying and wetting steps of dried nitrogen gas and 95 ± 0.5% RH air flow, respectively, and sample mass was monitored throughout the experimental procedure. Changes in measured mass were attributed to fiber drying and wetting, quantifying material hygroscopicity. The error in water vapor sorption was estimated using the propagation of uncertainty to be 0.52%.

## 3 Results and Discussions

### 3.1 Fiber Composition and Transmission

The BOFs used in this study are based on our recent report on MC-Alg composite optical fibers.^62^ MC is one of the simple chemically modified forms of cellulose, in which some of the hydroxyl (−OH) groups along the cellulose backbone are replaced with methoxyl (−OCH_3_) groups (Figure 1). This partial modification, typically characterized by a degree of substitution (DS) between 1.2 and 2.0, confers water solubility and a fully reversible lower critical solution temperature (LCST) behavior, distinct features not observed in unmodified cellulose.^52^ However, the properties of MC depend on several factors, including its molecular weight, concentration, degree of substitution, and the specific pattern of substitution along the polymer chain.^53^ Alg are linear polysaccharides composed of varying degrees of β-d-mannuronic acid (M) and α-l-guluronic acid (G) monomer units.^63^ The presence of carboxylate groups imparts a polyelectrolyte nature, making them readily water-soluble and sensitive to pH. MC and Alg are mutually compatible with complementary gelation behaviors, making them ideal for composite preparation through physical entanglement.^64^ At low temperature, MC polymer chains remain as random coils due to hydrogen bonding of residual −OH groups with water, resulting in a clear solution.^52^ Upon heating, the MC polymer chains transition from random coils to fibrillar aggregates, leading to gelation. On the other hand, Alg form gels in the presence of multivalent metal ions such as calcium (Ca^2+^). Therefore, MC-Alg composites allow fibers with enhanced mechanical performance, thermal stability and optical properties. In our previous study, we fabricated 21 combinations of MC-Alg composite fibers and demonstrated that these fibers are mechanically robust and show enhanced performance in the near-infrared (NIR) region.^62^ Our preliminary studies have revealed the rapid touch and humidity sensing properties of the fibers. This warranted us to explore the potential of these fibers for quantitative humidity sensing. We utilized wet spinning under ambient conditions for the fabrication of fibers. Unlike other fiber extrusion methods, wet-spinning is readily adaptable and compatible with biopolymers and their composites, as demonstrated for regenerated cellulose, Alg, MC, and carboxymethylcellulose under ambient conditions.^53,58,59,62^ The morphology, diameter, mechanical performances, and optical properties of fibers can be controlled by tuning the spinning dope concentration, flow rate, and coagulation conditions.^59,62^ Furthermore, spinning is performed under ambient conditions using aqueous coagulation conditions, resulting in reduced energy consumption and environmental impact. These attributes make wet-spinning a method of choice for readily scalable biopolymer fibers for diverse applications. Accordingly, we used the fibers fabricated using wet-spinning with the following compositions: (i) one-component fibers consisting of either MC with a molecular weight of 88 000 g/mol (MC88) or Alg (ii) and two-component systems consisting of MC-Alg (see details in the Supporting Information). The fiber extrusion was performed using an optimized spinning dope with an overall solid content of 4%, and a flow rate of 1.8 mL/min. Two types of coagulation conditions, namely a 96:4 ethanol:water (v/v) mixture and ionic coagulation, were employed with a flow rate of 1.8 mL/min. For Alg and Alg containing composites, ionic coagulation was performed using aqueous calcium chloride. The scanning electron microscopy (SEM) images revealed smooth surface morphology of all fibers (Figure 1g,h). However, the ethanol coagulated MC fibers show flattened geometry (Figure S4). Whereas the ionically coagulated Alg and MC-Alg fiber displayed circular morphology with lateral diameters of fibers in the range of 100–300 μm (Figure 1g,h). Ionic coagulation is instantaneous, allowing fibers with a circular cross-section. This results in rapid alignment and crystallization of polymer chains. On the other hand, ethanol is a softer coagulant, and the exchange between water and ethanol occurs at a slower rate. This allows the fibers to flatten against the coagulation bed, resulting in a more elliptical cross-section. This slow exchange leads to a more gradual and less uniform phase separation, resulting in less dense and more amorphous fiber structures. It is well documented in the literature that ethanol coagulation significantly reduces the crystallinity and molecular orientation of the fibers. The cross-sectional circularity in fibers was evaluated as the ratio of the cross-sectional area of the fiber *A* to the square of the perimeter of the fiber cross-section *P* as follows:

2 $$\theta =\frac{4\pi A}{P^{2}}$$

where *θ* is the dimensionless circularity percentage parameter.^65^ Fiber cross-section visualization is available in SEM images in Figure S4. We have identified a combination of four fiber samples suitable for quantitative humidity sensing. Table 1 summarizes the compositions, methods, and key optical properties of the BOFs.

**Table 1:** Compositions, Fiber Fabrication Methods, and Properties^a^

Material composition	MC 4%	Alg 4%	MC-Alg 3/1%	MC-Alg 3/1%
Coagulation method	EtOH	CaCl_2_	EtOH	CaCl_2_
*D* (μm)	230 – 270	200 – 230	200 – 230	230 – 270
θ (%)	75.79	98.35	72.95	99.99
*n*_633_	1.4907	1.5216	1.4984	1.4984
*T* (nm)	500 – 1170, 1240 – 1340	750 – 1190, 1230 – 1320	700 – 1350	700 – 1180, 1220 – 1340
*α*_770 nm_ (dB cm ^–1^)	4.14 ±0.10	6.91 ±0.17	7.94 ±0.20	5.65 ±0.14
*α*_1310 nm_ (dB cm^–1^)	4.33 ± 0.14	8.85 ± 0.30	6.87 ± 0.23	4.19 ± 0.14
*S*_Vis_ (dB/%RH)	0.152 ± 0.005	0.037 ± 0.001	0.331 ± 0.011	0.04 ± 0.001
*λ*_Vis_ (nm)	903	905	902	902
*S*_NIR_ (dB/%RH)	–0.089 ± 0.003	–0.056 ± 0.002	0.205 ± 0.007	–0.056 ± 0.002
*λ*_NIR_ (nm)	1353	1353	942	1353

a Summary of fiber diameter *D*, circularity *θ*, refractive index at 633 nm *n*_633_, transmission window *T*, attenuation at 770 nm α_770*nm*_ and 1310 nm α_1310*nm*_, humidity sensitivity peaks at Vis (*S*_Vis_) and NIR (*S*_NIR_) region and the respective peak wavelengths, λ_Vis_ and λ_NIR_.

The transmission spectra of the fibers were measured between 400 and 1700 nm, using a UV–vis spectrometer (400–1100 nm) and an NIR spectrometer (900–1700 nm). The MC fibers show good transmission above 400 nm, whereas Alg and MC-Alg composite fibers display transmission above 700 nm. Importantly, Alg fibers show lower transmission at the visible and NIR regions compared to that of the MC fibers (Figure S5) . Furthermore, MC-Alg composite fibers display transmission between one-component MC and Alg fibers.

### 3.2 Humidity Sensitivity of One-Component BOFs

The attenuation values of one-component MC and Alg fibers were measured using the cutback method (Figure 2a). Photon count spectra are recorded with the spectrometer and converted into logarithmic losses spectra using eq 1 to make them comparable with each other and other literature (see original count spectra in Figure S6). The visible region attenuation has been reported in our previous work at 770 nm (edge of red light) to ensure comparability between sample types as Alg fibers transmit poorly below 750 nm,^62^ while the NIR attenuation is reported at 1310 nm for easier literature comparison as it is widely used as the center wavelength of the telecom O-band optimized for single mode fibers.^66^ Applying the Beer–Lambert fitting (see Experimental Section) to each wavelength of the spectral data provides an estimation of the optical losses with high linearity on the logarithmic scale (Figure 2b). The fitting was performed at 900 nm for the visible region and 1310 nm for the NIR region. MC fibers exhibit attenuation values of 4.14 dB cm^–1^ and 4.34 dB cm^–1^, respectively, in the visible and NIR region (Figure 2b and Table 1).^62^ On the other hand, the attenuation values of Alg fibers were 6.91 dB cm^–1^ and 8.85 dB cm^–1^, respectively, in the visible and NIR region.

**Figure 2:**
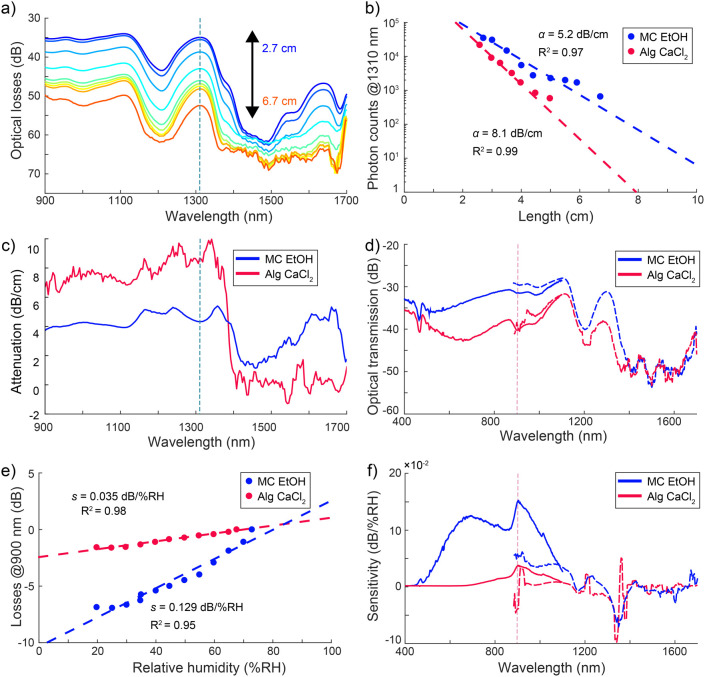
Optical properties and humidity sensitivity of one-component fibers. (a) NIR transmission spectra from the MC fibers measured using the cutback method. (b) Examples of Beer–Lambert fitting to the spectral data with high linearity on the logarithmic scale to determine the attenuation. (c) The attenuation spectra with the lowest reliable losses around 1100, 1300, and 1600 nm. (d) UV–Vis (solid line) and NIR spectra (dashed line) recorded from the MC and Alg fiber samples show minimum losses around 1100 nm. (e) Representative graph showing linear regression from the MC and Alg spectra in normalized losses per RH to determine (f) the humidity sensitivity spectra in UV–Vis (solid line) and NIR (dashed line) showing the highest RH response in the 600–1000 nm range with low sensitivity above 1000 nm but a linear response to humidity.

Attenuation spectra revealed possible water peaks, as distinct peaks are prominent in the attenuation spectrum of Alg fibers at wavelengths of 921, 1163, 1255, and 1342 nm (Figure 2c). Similar but more moderate increases in attenuation can be seen in the attenuation spectrum of MC fibers at 1167, 1239, and 1357 nm. Consequently, local attenuation minima for MC fibers are located around 1100 and 1300 nm. Similar but less pronounced attenuation minima can be observed for Alg fibers at around 1120 and 1305 nm. To investigate the humidity response, optical loss measurements were performed by exposing the BOFs to varying %RH in a custom-made experimental chamber (Figure 1d). UV–vis and NIR transmission spectra were collected periodically by varying the RH in the range from 20 to 80% RH. Figure 2d shows the representative transmission loss spectra of ethanol coagulated MC fiber and CaCl_2_ coagulated Alg fiber measured using a UV–vis spectrometer (400–1100 nm, solid lines) and a NIR spectrometer (900–1700 nm, dotted lines) under controlled humidity. In the visible spectral region, the BOFs displayed peak humidity sensitivity at 900 ± 4 nm, while sensitivities in the NIR region were around 1357 ± 5 nm (Table 1). Linear regression fitting of optical losses versus RH revealed high humidity sensitivity up to 0.152 dB/%RH at 903 nm in MC fibers with 4.64% hysteresis and 20 s response time. In contrast, the sensitivity drastically decreases above 1000 nm (Figure 2e). Above 1300 nm, no linearity in the humidity response is observed, as the sensitivity spectrum decays into noise (see Supporting Information S7 and S8 for the coefficients of determination for the attenuation and humidity sensitivity spectra, respectively). A similar trend can be observed in Alg fibers: humidity sensitivity starts at around 750 nm, with a sensitivity peak of 0.037 dB/%RH at 905 nm, exhibiting 2.94% hysteresis and a response time of 60 s. Curiously, a higher but inverse sensitivity is achieved near the hydroxyl ion absorption region at 1353 nm. In this region, an increase in RH leads to lower transmission at a sensitivity rate of 0.089 dB/%RH for MC fibers and 0.056 dB/%RH for Alg fibers. Overall, the peak humidity sensitivity of Alg fibers is nearly three times lower than for MC fibers in the visible region of the spectrum. Transmission changes over multiple cycles of humidity ramping at select wavelengths are further illustrated in Figures S9 and S10. After the first-cycle hysteresis, the humidity response was reproducible in subsequent cycles, and transmission recovered to its initial level, indicating good long-term stability under cyclic humidity exposure. Furthermore, in our previous work, we have demonstrated that that MC-Alg BOFs show minimal changes in optical properties after 1050 days of storage on the shelf in controlled laboratory conditions.^62^

### 3.3 Humidity Sensitivity of Composite BOFs

Attenuation spectra of ethanol coagulated MC-Alg composite fibers showed attenuation of 7.94 dB cm^–1^ and 6.87 dB cm^–1^, respectively, in the visible and NIR region (Figure 3a–c and Table 1). On the other hand, ionically coagulated MC-Alg fibers show attenuation of 5.65 dB cm^–1^ and 4.19 dB cm^–1^, respectively, in the visible and NIR region (Figure 3). Overall, the composite BOFs exhibit slightly higher attenuation than that of one-component MC fibers, but significantly lower than that of one-component Alg fibers.

**Figure 3:**
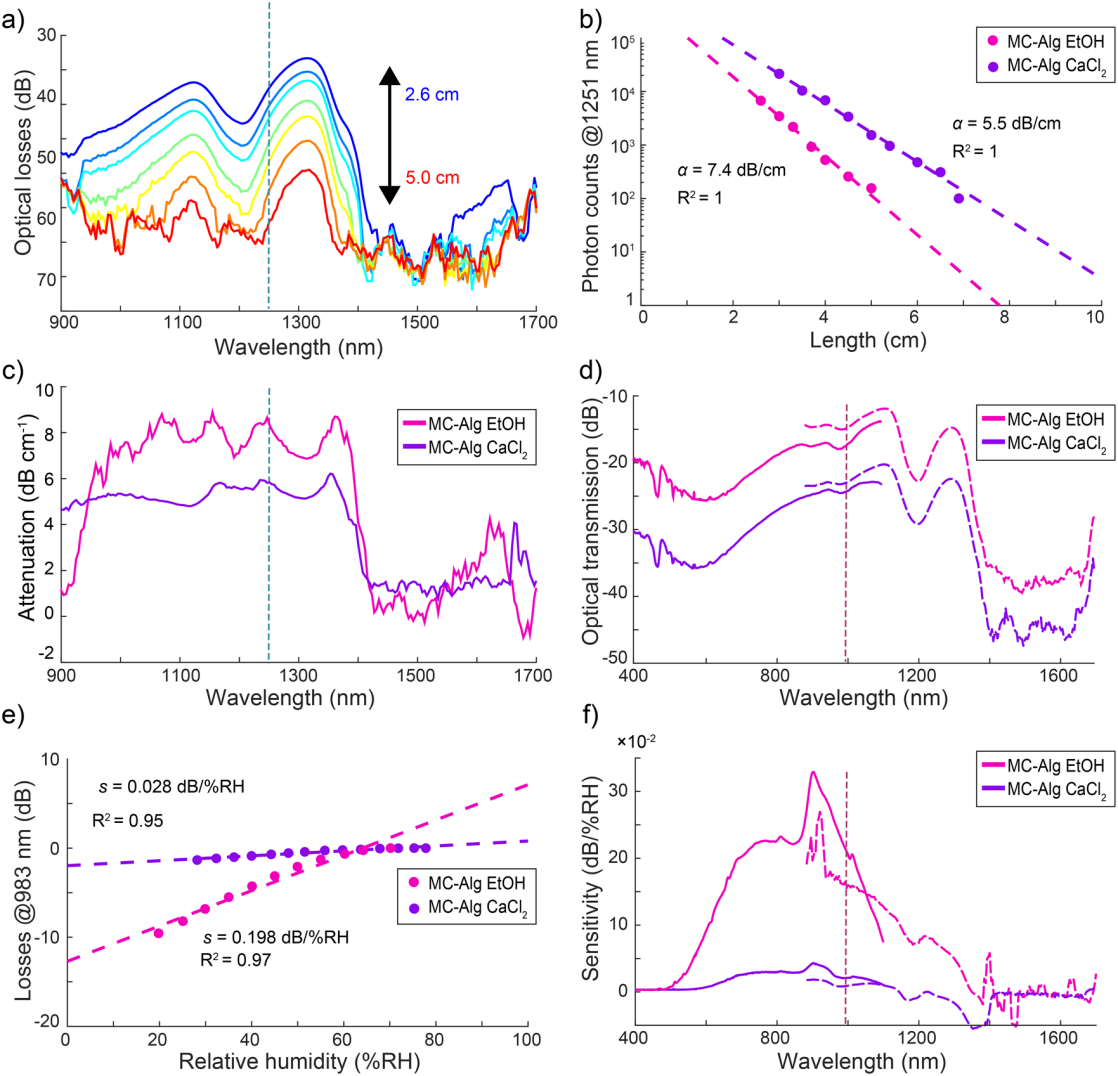
Optical properties and humidity sensitivity of MC-Alg composite fibers. (a) NIR transmission spectra of ethanol coagulated MC-Alg fiber from the cutback method. (b) An example of Beer–Lambert fitting to the spectral data with high linearity on the logarithmic scale to determine. (c) The attenuation spectra with consistent 5–8 dB cm^–1^ attenuation across the 900–1400 nm range. Minimal attenuation is achieved with ionic coagulation. (d) UV–Vis (solid line) and NIR spectra (dashed line) of fibers show minimum losses around 1100 and 1300 nm, with the least losses recorded from MC-Alg composite fibers. (e) An example of linear regression to normalized losses from the MC-Alg EtOH fiber spectra per RH to determine humidity sensitivity. (f) The humidity sensitivity spectra show the highest response in the 800–1000 nm range with low but linear sensitivity above 1000 nm.

The transmission spectra of composite fibers display similar features in their optical losses, but in between MC and Alg fibers (Figure 3d). Notably, the ethanol-coagulated composite fibers also exhibit a high humidity sensitivity of 0.33 dB/%RH at 902 nm, with 11.1% hysteresis and a 90 s response time, a sensitivity that is nearly twice that of one-component MC fibers (Figure 3e,f). The presence of Alg extends the humidity sensitivity region of ethanol-coagulated BOFs nearly to 1300 nm. On the other hand, in one-component MC fibers, the sensitivity decreased beyond 1100 nm. Despite having lower optical losses, ionically coagulated MC-Alg fibers neither exhibit high humidity sensitivity nor a wide sensitivity wavelength range. Their humidity sensitivity peaks around 0.05 dB/%RH at 910 nm, with 2.28% hysteresis and a response time of 120 s. Notably, the ethanol-coagulated MC-Alg composite fiber exhibits the highest humidity sensitivity, approximately six times higher than its ionically coagulated composite BOFs. Normalized transmission changes of ethanol-coagulated MC-Alg fibers in the NIR region under repeated RH cycles revealed that humidity sensitivity is repeatable with hysteresis of up to 6 dB or 11.1% (Figure S11). Furthermore, the ethanol coagulated MC and MC-Alg fibers showed similar transmission around 1300 nm. While ionically coagulated Alg and MC-Alg fibers, as well as ethanol-coagulated MC fibers, exhibit negative humidity sensitivity around 1353 nm. A similar humidity sensitivity is observed only for ethanol-coagulated MC-Alg at 1396 nm, with both wavelengths residing close to the hydroxyl absorption band (see Figures S12 and S13).

### 3.4 Dynamic Vapor Sorption Analysis of BOFs

To gain insights into humidity sorption in BOFs, we employed DVS experiments. DVS is a gravimetric analytical technique that allows precise measurement of the rate and quantity of solvent vapor absorbed by the sample under controlled experimental conditions.^67–70^ In a typical experiment, sample fibers were placed on a microbalance in a chamber flooded with alternating intervals of dry nitrogen gas and 95% RH air to monitor the saturated BOF water intake. Samples were first dried for 12 h at 0% RH under a nitrogen flow to remove any moisture from storage conditions. All samples presented in Figure 4 reached their saturated dry mass within the first 3 h. Subsequently, each sample was exposed to 72 h of 95% RH airflow, and its mass change was monitored. MC fibers achieved near-saturation mass (>90%) within 3 h of exposure to humid air and could absorb up to 80% of their mass in water. Similar results were obtained through monitoring optical transmission through MC fibers during extended exposure to a high RH environment, where the optical signal saturated after 6 h (see Figure S14). However, for Alg fibers, the water intake was much slower and did not reach a saturation point during the 72 h high RH step.

**Figure 4:**
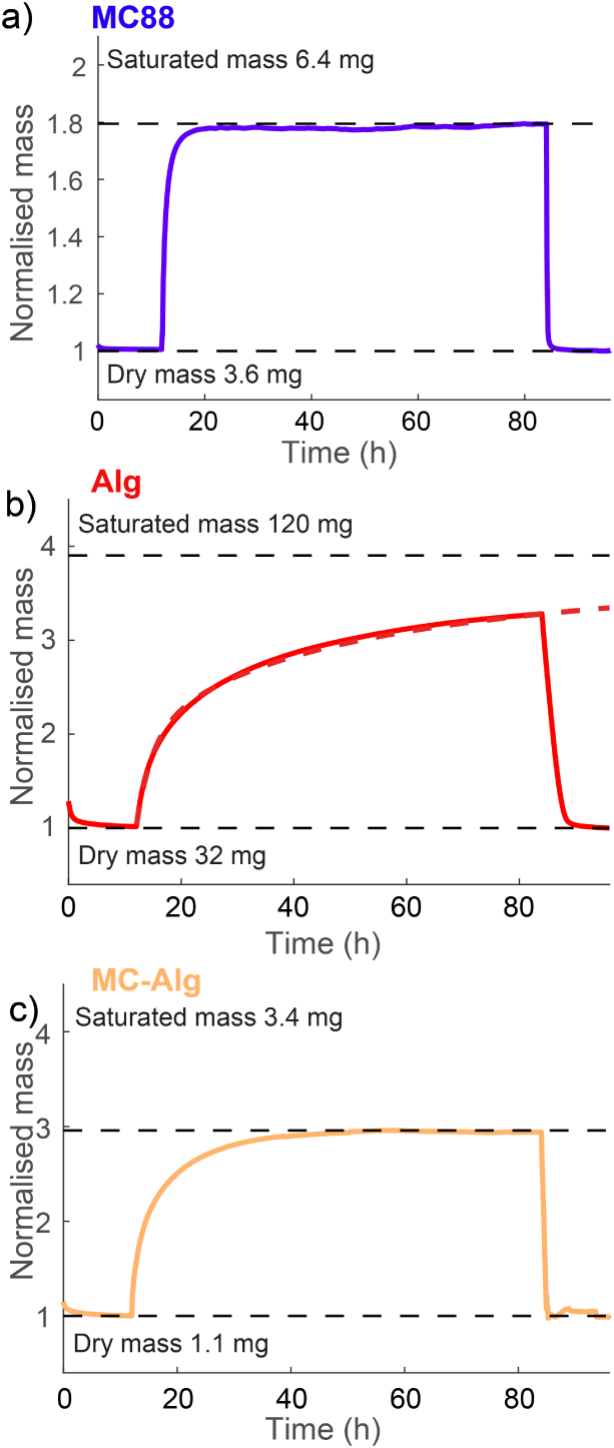
Hygroscopicity in DVS measurements. BOF samples were exposed to humidity ramping of 12 h at 0% RH (dried N_2_ gas), 72 h at 95% RH, and 12 h at 0% RH again. (a) MC fiber reaches hygroscopicity equilibrium within 6 h of exposure to 95% RH at a 0.31 mg/h rate, absorbing 80% of its dry mass in water. Desorption occurs notably quicker, within 1 h. (b) Alg fibers exhibit the greatest but also slowest moisture absorption, absorbing nearly four times their initial dry mass in about 200 h at 1.2 mg/h. Its drying is also slower than that of other studied BOFs, taking over 6 h to reach dry mass after 72 h of exposure to 95% RH. (c) MC-Alg composite fiber exhibits a combination of features of faster absorption from MC and higher water intake of Alg, absorbing moisture faster and reaching its 300% dry mass equilibrium in 24 h at 0.68 mg/h rate. The drying cycle is notably faster, with dry mass reached within 1 h.

Although the studied Alg samples were heavier than other BOF samples, their normalized mass change was also greater, with water intake surpassing the dry mass by over 2-fold during the moisture exposure. The total saturated mass was estimated by fitting an approximation of the moisture sorption isotherm based on the monitored curve. As water molecules absorb into BOFs through strong monolayer adsorption, the Brunauer Type I moisture sorption isotherm can be used to estimate the saturated mass.^69^ Here, a two-term exponential function was found to provide the best fit:

3 $$m=a_{1}\;e^{b_{1}t}\cdot a_{2}\;e^{b_{2}t}$$

where, *a_n_* and *b_n_* are fitting parameters with mass *m* varying as a function of time *t*. The estimated saturated mass for Alg fibers is nearly three times the dry mass. Alg also affects the water sorption rate in composite fibers (Figure 4c). For example, when 1 g of MC-Alg (3 + 1 m%) fiber was exposed to 95% RH for 72 h, reaching near-saturation mass took over 20 h, with only 25% of the BOF mass being Alg. Water intake was also higher for the MC-Alg composite fiber, reaching up to 200% of its dry mass.

Desorption behavior is similar to that of other cellulose-based materials, with faster water desorption than adsorption.^71^ Upon exposing the saturated wet weight samples to dry N_2_ gas flow, one-component MC fiber samples reached near-dry mass (<10%) within 20 min. At the same time, similar drying took over 200 min for Alg fibers. MC-Alg composite fiber desorption behavior was a hybrid of MC and Alg fibers; near-dry mass was achieved in 50 min, significantly faster than for one-component Alg fibers but still over twice as slow as for MC fibers. Alg contains a large number of carboxylate (−COO^–^) and hydroxyl (−OH) groups facilitating strong hydrogen bonding with water molecules. This results in significantly high affinity for moisture update during DVS measurements. On the other hand, in the case of MC, some of the hydroxyl groups are substituted with methoxy (−OCH_3_) groups. Therefore, the overall number of hydrophilic groups for hydrogen bonding with water are less compared to alginate. Consequently, MC fibers display lower moisture uptake than that of Alg fibers. However, in MC-Alg composite fibers, the carboxylates and hydroxyl group of alginates are embedded in the MC matrix, resulting in rapid and high moisture uptake. Based on these water sorption isotherms, MC produces the fastest RH sensor response times while Alg can produce the greatest dynamic range for measurements. The optimal solution for RH sensing appears to be an MC-Alg composite fiber, where the alginate content of just 25% of the BOF mass already increases water intake by 150% compared to pure MC fibers, while still maintaining reasonable adsorption/desorption times. BOF response times to changes in humidity were evaluated by monitoring transmission through the fiber under fluctuating humidity. Response times are comparable to those of the commercial Linkam sensor used for humidity control, as shown in Figure S3.

### 3.5 Effect of Temperature on the Humidity Sensitivity of BOFs

Thermal sensitivity in BOFs and its impact on their humidity sensitivity were studied in the 20–100 °C temperature range (Figure 5). Fiber samples were heated using a standard heat plate and allowed to cool to room temperature while their transmission in the UV–vis spectral region was monitored. A decrease in transmission during heating can be observed in all BOFs.

**Figure 5:**
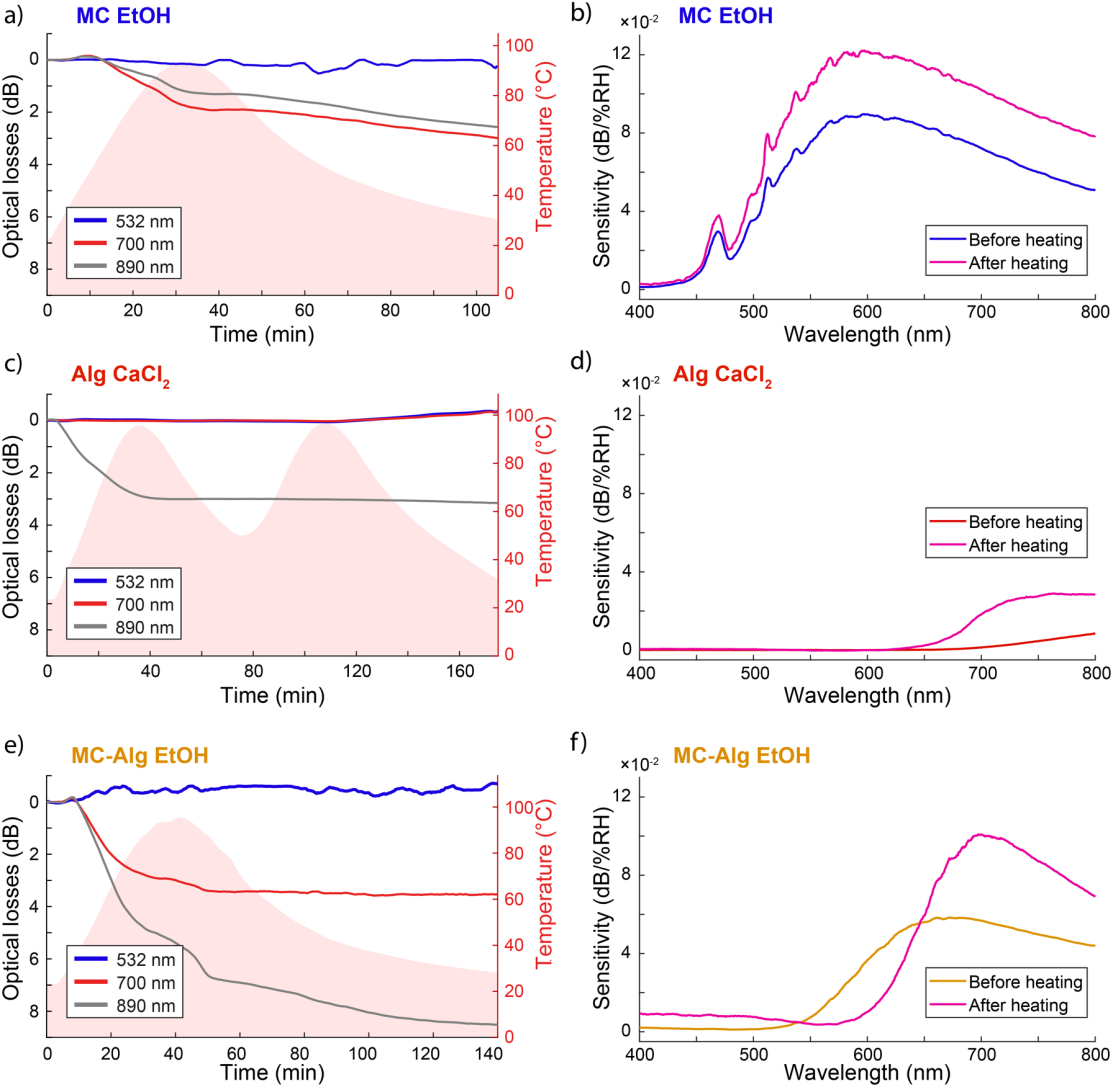
Effect of heating on humidity sensitivity of BOFs. The optical losses of the BOFs are presented at different wavelengths during a heating–cooling cycle. The humidity sensitivity was measured before and after heating the fibers. (a,b) Optical loss and humidity sensitivity of ethanol coagulated MC fibers. (c,d) Optical loss and humidity sensitivity of ionically coagulated Alg fibers, and (e,f) Optical loss and humidity sensitivity of ethanol coagulated MC-Alg composite fibers.

However, the changes in transmission do not follow the same trend as changes in temperature (Figure 5). Therefore, the fibers are not notably sensitive to temperature directly, but rather exhibit reduced transmission due to moisture evaporation.^62^ Heating BOFs evaporates absorbed water, promoting crystallinity and reducing the GI-BOF nature of water-containing BOFs, resulting in lower transmission. MC fibers exhibit an additional 2–3 dB transmission loss throughout the heating–cooling cycle, indicating significant water evaporation but no apparent temperature sensitivity (Figure 5a). Alg fibers naturally transmit poorly below 800 nm and thus, no significant changes were observed at 532 or 700 nm. However, the NIR transmission at 890 nm decreased quickly by 3 dB within the first 40 min. Notably, further changes in temperature do not affect the transmission in the second heating and cooling cycle (Figure 5c). MC-Alg composite fibers exhibited the greatest response to heating, with a 700 nm transmission dropping by almost 4 dB and an 890 nm NIR transmission of over 8 dB (Figure 5e). This suggests high initial moisture content in the MC-Alg composite fibers. The changes in transmission, however, did not reveal any clear thermal sensitivity beyond the evaporation of absorbed water. Thermal ramping also heightened the BOF humidity sensitivity as the fibers were effectively dried. Therefore, greater changes in transmission could be observed upon water sorption. This suggests that the BOF sensors can be exposed to temperatures up to 100 °C without loss of sensitivity. For MC fibers, the humidity sensitivity increased by up to 60% between 500 and 800 nm (Figure 5b), while Alg fiber sensitivity increased by almost 5-fold. The humidity sensitivity peak for Alg fibers before heating was an order of magnitude lower than for MC fibers, and still, after heating, four times lower (Figure 5d). However, for MC-Alg composite fibers, the humidity sensitivity doubled below 540 nm and above 650 nm but decreased substantially in between. Importantly, the individual components of the composite fiber are, on their own, sensitive to humidity above 650 nm. The sensitivity increase in this range is close to the average of its components, with the sensitivity peak at 0.100 dB/%RH around 700 nm. Evaluating the thermal sensitivity of bulk samples will unambiguously address whether dehydration or intrinsic material sensitivity is responsible for the residual losses observed in the first heating cycle. However, bulk samples of biopolymers often result in aggregation and opacity, making transmission-based measurements challenging. Thin films are a potential alternative for thermal analysis, but with much shorter path length compared to fibers. The temperature sensitivity was studied using MC, Alg and MC-Alg composite thin films with repeated heating–cooling cycles from 25 to 100 °C (see for details and Figure S15). Thin films exhibit only limited temperature sensitivity (0.1–0.7 dB transmission change) across 25–100 °C, with dehydration during heating as the predominant mechanism. MC films display slight irreversible transmission loss, Alg films behave reversibly, and MC–Alg composite films show the highest reversible change, demonstrating that material composition governs both magnitude and reversibility of optical response. According to our previously reported differential scanning calorimetry and thermogravimetric analysis results, the studied BOFs are thermally stable and only begin to decompose around 250 °C.^62^

### 3.6 Comparison of the Performance of Different Humidity Sensors

The BOFs studied in this work do not have clear core-cladding structures. More importantly, their rich surface chemistry and the presence of hydrophilic functional groups offer rapid sorption and resorption of solvent vapors. Therefore, the surrounding environment, such as humidity, acts as a dynamic surface refractive index modifier. This allows for an altered core-cladding refractive index, which affects light transmission in response to changes in environmental conditions.

A wealth of literature reviews on optical and cellulose-derived humidity sensor systems have been published in recent years.^6,11,12,72–74^ A literature summary of various resistive, capacitive, and optical humidity sensors published in the past decade is presented in Figure 6. Humidity sensor systems are compared based on their sensitivity (dB/%RH) and sustainability, in terms of biopolymer content in sensor design. Based on this summary, no fully biobased optical humidity sensors have been quantitatively characterized, and even the more biobased sensor systems contain at most 50% biopolymer materials. Here, biopolymer content was evaluated based on sensor material design, excluding the signal sources (light or voltage) and analyzers (photodetector or meter). For cases where biopolymer materials were used as sensor substrate but not the main sensing component, e.g., electronics printed on cellulosic paper, biopolymer content was evaluated at 25%, while for designs combining biopolymer materials with other materials in sensor elements at an unspecified but roughly equal proportions, biopolymer content was evaluated at 50%. Given that the vast majority of sensor designs contain no biopolymer content, the presented examples from the literature with 0% biopolymer content are limited to a few representations (see Table S1 for the entire list of references).

**Figure 6:**
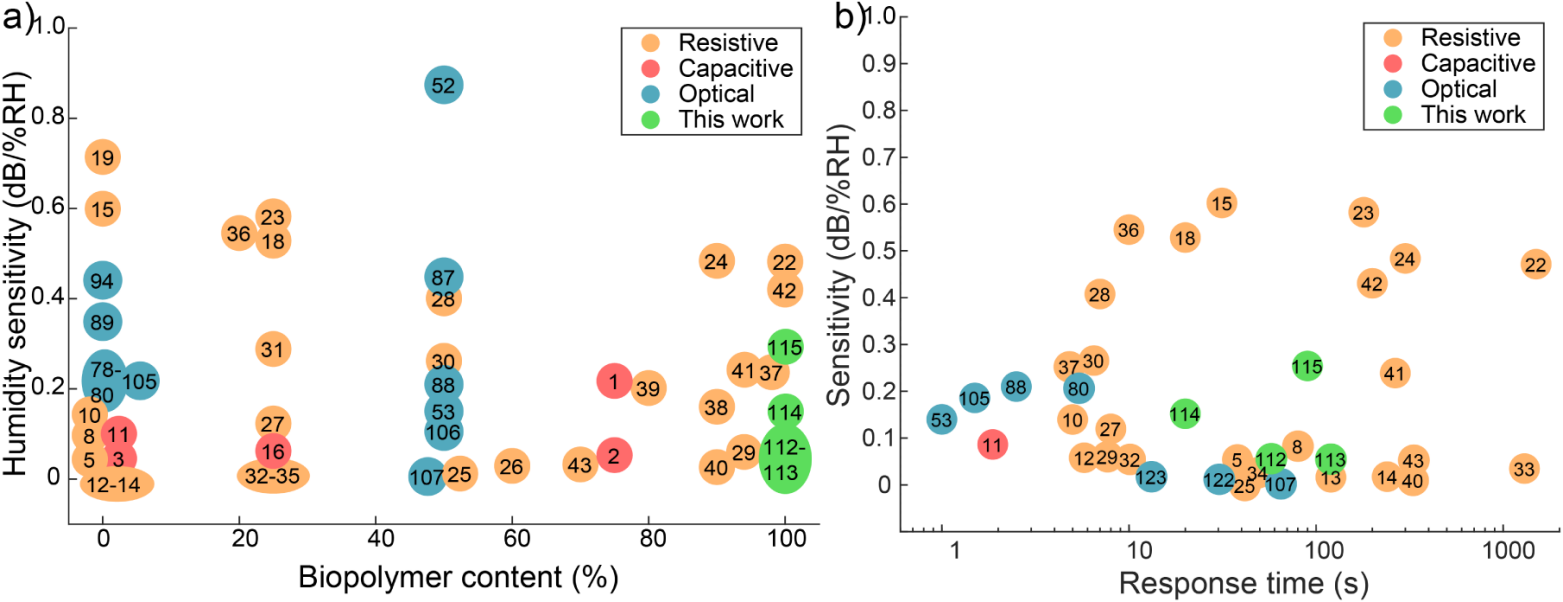
Comparison of humidity sensors. (a) A comparison of the literature values for humidity sensitivity in resistive, capacitive, and optical sensor systems by device biopolymer content published in the past 10 years. (b) A comparison of sensitivity vs response time reveals the first fully biopolymer optical sensor systems with quantitatively characterized humidity sensitivity studied in this work.

BOF hysteresis in RH measurements varied between 2 and 11%. Ionically coagulated Alg and MC-Alg BOFs displayed lower hysteresis of 2.94% and 2.28%, respectively, while MC and MC-Alg BOFs coagulated in ethanol exhibited much greater hysteresis of 4.64% and 11.1%, respectively. As vapor sorption kinetics in ionically coagulated BOFs were an order of magnitude slower, higher hysteresis for ethanol-coagulated samples during 15–20 h RH ramping can be attributed to faster vapor sorption. A comparison of the literature values for hysteresis and humidity sensitivity in resistive, capacitive, and optical sensor systems is presented in Figure S16.

Our analysis revealed that the ethanol coagulated MC-Alg BOFs are equally or more sensitive than most of the compared literature values for optical sensors (Figure 6a). With a RH sensitivity of 0.331 dB/%RH, the BOF sensitivity also exceeds that of reported capacitance-based RH sensor designs. It is also noteworthy that the majority of the higher-sensitivity optical sensor designs involve operating at a limited humidity range such as 84–95% RH (94), 60–77% RH (89), or 60–90% RH (80), or rely on bulky and expensive equipment such as optical spectrum analyzers (52, 79, 87, 88, 105). While some biobased resistive humidity sensors provide equal or greater sensitivity to BOF sensors reported here, there are applications where resistance-based sensing is not feasible, such as areas with high electromagnetic interference or medical applications that require biocompatible, nontoxic materials.

## 4 Conclusions

In recent years, there has been considerable interest in the fabrication of optical fibers made from both synthetic polymers and naturally derived biopolymers. While previous reports in the literature have demonstrated their potential for passive sensing applications, realizing fully biopolymer-based optical fibers capable of performing quantitative sensing of environmental parameters has remained a challenge. Among biopolymers, polysaccharides such as as methylcellulose and alginates have emerged as promising candidates due to their unique combination of chemical versatility, mechanical flexibility, optical properties, and biodegradability. Their inherent compatibility, unique gelation properties and tunable refractive indices offer new avenues for the fabrication of biobased composite optical fibers tailored for short-distance applications and environmental sensing. In this work, for the first time, we demonstrated that quantitative humidity sensing can be performed using optical fibers made of biopolymers comprising methylcellulose and alginate. The fiber morphology and humidity sensitivity can be tuned by adjusting the fiber composition or coagulation conditions. By leveraging the inherent hygroscopic properties and optical responsiveness of methylcellulose and alginate, the studied BOFs allow measurable and reproducible changes in their optical response to changing relative humidity levels with a sensitivity up to 0.30 dB/%RH. The observed sensitivity is equal to or better than the low cost capacitive or resistive humidity sensors. Furthermore, the fiber composition and fabrication methods provide wavelength-specific sensitivity, spanning a wide wavelength range from the visible to the near-infrared regions. Unlike low-cost electronic sensors, the humidity sensing is independent of temperature, making them suitable for measuring various environmental parameters under fluctuating temperature conditions. Our finding highlights the feasibility of natural polymers as a sustainable and effective platform for quantitative humidity sensing. The findings presented in this work lay the foundation for future bio-optical sensing platforms that are scalable and adaptable for a wide range of applications, including environmental monitoring, wearable diagnostics, and smart packaging. Further optimization of the fiber fabrication process and data acquisition methods could enable enhanced sensing, response time, and long-term stability. Additionally, integrating these fibers into miniature devices could accelerate their translation into real-world applications. Overall, our work represents a crucial step toward the sustainable sensing of environmental parameters using natural polymer-based optical fibers.

## Supplementary Material



## Notes

NIR attenuation measurements, humidity sensitivity of PMMA, humidity sensitive spectra of BOFs, humidity response time of BOFs, scanning electron microscopy (SEM) images, normalized NIR spectra of BOFs, BOF transmission spectra, NIR attenuation spectra of BOFs, Vis transmission spectra of BOF during RH ramping, NIR transmission spectra of BOF during RH ramping, RH hysteresis of BOFs, and a comparison of humidity sensors (PDF)

## #

Espatent Oy, Kaivokatu 10D, 00100 Helsinki, Finland

## Abbreviations

MC: Methylcellulose
Alg: Alginate
SEM: Scanning Transmission Electron Microscopy
DVS: Dynamic Vapor Sorption
NIR: Near-Infrared
RH: Relative Humidity
UV–vis: Ultraviolet–Visible
