# Supplementary material for: Biopolymer Optical Fibers for High-Sensitivity Quantitative Humidity Monitoring
Source: ACS Appl Mater Interfaces. 2025 Aug 25;17(35):49816–28. doi: 10.1021/acsami.5c10056 (PMC12412099; doi:10.1021/acsami.5c10056)
Supplement: Supplementary file 1 [file am5c10056_si_001.pdf]

Supporting Information

Biopolymer Optical Fibers for High-Sensitivity Quantitative Humidity Monitoring

Jani Patrakka,[a] *Ville Hynninen,*[a,†] *Petteri Huttunen,*[b] *and Nonappa*[a,*]

[a]Faculty of Engineering and Natural Sciences, Tampere University, Korkeakoulunkatu 6, FI-33720, Tampere, Finland

[b]Faculty of Built Environment, Tampere University, Korkeakoulunkatu 5, FI-33720, Tampere, Finland

*Corresponding author: nonappa@tuni.fi

Table of Contents

1. Materials…………………………………………………………………….S3

2. Near-infrared (NIR) attenuation measurements………………………………S3-S4

3. Figure S1. PMMA humidity sensitivity……………………………………...S5

4. Figure S2. Converting photon counts into BOF humidity sensitivity spectra….S6

5. Figure S3. BOF humidity response time……………………………………..S7

6. Figure S4. Scanning electron microscopy images of BOFs…………………….S8

7. Figure S5. Normalized NIR spectra of BOFs…………………………………S9

8. Figure S6. BOF transmission spectra………………………………………S10

9. Figure S7. BOF NIR attenuation spectra……………………………………..S11

10. Figure S8. BOF humidity sensitivity spectra……………………………….S12

11. Figure S9. BOF Vis transmission during RH ramping…………………………..S13

12. Figure S10. BOF NIR transmission during RH ramping……………………… .S14

13. Figure S11. MC-Alg EtOH RH hysteresis………………………………… S15

14. Figure S12. BOF humidity sensitivity at 1353 nm……………………….....S16

15. Figure S13. MC-Alg EtOH humidity sensitivity at 1353 nm and 1396 nm…….S17

16. Figure S14. MC BOF in high humidity…………………………………… S18

17. Figure S15. Humidity sensitivity of thin films……………………………..S19

16. Figure S16. Comparison of humidity sensors……………………………….S20

17.Table S1. List of literature references used for comparative study………………S21-S23

1.0 Materials

The biopolymers were acquired from commercial sources. Methylcellulose (MC), sodium alginate (Alg) and $CaCl_2$, were purchased from Sigma Aldrich and were used as received. Ultrapure Milli-Q® water (Type I, 18 MΩ.cm) was used for all experiments. MC polymers with molecular weights (MW) 88000 g mol^{-1} with a degree of substitution (DS) of 1.5–1.9 were used were used. The degree of substitution is defined as the average number of $-CH_3$ groups per monomer unit at a maximum of 3.

2.0 Near-Infrared (NIR) attenuation measurements

The attenuation coefficient was estimated with the cutback method. NIR spectrometer was used to record transmitted white light from a halogen lamp (Ocean Optics DH-2000-BAL, 230 – 2500 nm). The attenuation coefficient, α can be determined numerically from a dataset of light power and fiber length data pairs by applying the Beer-Lambert law.

$$P(L_i) = P_0\, e^{-\alpha L_i} \qquad \text{(S1)}$$

WhereP_0 is the input power, $P(L_i)$ is the output power at length L_i, and α is the attenuation coefficient. Applying the Beer-Lambert law involves the assumptions of constant input power and coupling efficiencies. Input and output powers can also be converted into logarithmic transmission values in dB to simplify the attenuation dependence on length into a linear equation

$$P(L_i) = P_0 - \alpha L_i \qquad \text{(S2)}$$

Attenuation in sample BOFs was determined at 208 wavelengths between 900 – 1700 nm. White light was coupled into a commercial multimode fiber (Thorlabs M12L01, Ø300 µm, 0.39 NA), and the samples were butt-coupled to the commercial multimode fiber. Transmitted light was

measured with an Avantes AvaSpec-NIR256-1.7-EVO spectrometer (900 – 1700 nm operating range with InGaAs detector and 200 lines/mm grating, 4 nm resolution and down to 10 μs integration time). Samples were prepared by cutting them into a suitable initial length (3 – 6 cm) and transmitted light was measured incrementally from the sample as it was cut shorter. Fiber length was determined with a standard metric ruler for each increment. The photon counts and fiber length data were analyzed with MATLAB to resolve input power, coupling losses and the attenuation coefficient for each wavelength using numerical fitting. Error in attenuation was estimated using propagation of uncertainty

$$\sigma_\alpha \approx \alpha\sqrt{\left(\frac{\sigma_I}{I}\right)^2 + \left(\frac{\sigma_L}{L}\right)^2} \qquad \text{(S3)}$$

where the standard deviation of attenuation σ_α can be evaluated with relative errors from the spectrometer $\frac{\sigma_I}{I}$ and length measurements $\frac{\sigma_L}{L}$. As the UV-Vis and NIR spectrometers have 0.02 nm and 0.42 nm accuracies at the measured wavelength range, respectively, and the error in length measurement is estimated to be 0.1 cm, the errors in attenuation were calculated to be at most 3.3 %.

3.0 Biopolymer thin film temperature sensitivity measurements

Biopolymer spinning dopes described in Experimental Section 2.2 – 2.4 were drop cast into thin films to study their temperature sensitivity. To remove surface contaminants, standard glass microscope slides (75 by 26 mm) were cut in half and sonicated (Elma Elma-sonic P sonicator) at 37 kHz and 30 °C for 20 min in three solutions: acetone, isopropyl alcohol, and Milli-Q water. After the third sonication, excess water was removed with nitrogen gas flow. Cleaned microscope

slide pieces were then placed in petri dishes and 2 mL of biopolymer spinning dope of each type was poured onto them. The petri dishes were left to dry in ambient lab conditions overnight, and the resulting biopolymer-coated microscope slides were measured in the Linkam LTS420-H humidity controller chamber. Three cycles of heating and cooling (25 to 100 ˚C) were performed on each biofilm in controlled 50 ± 5 %RH humidity, and light transmission through the films was monitored during the temperature ramping using the same equipment as specified in Experimental Section 2.7 – 2.8.

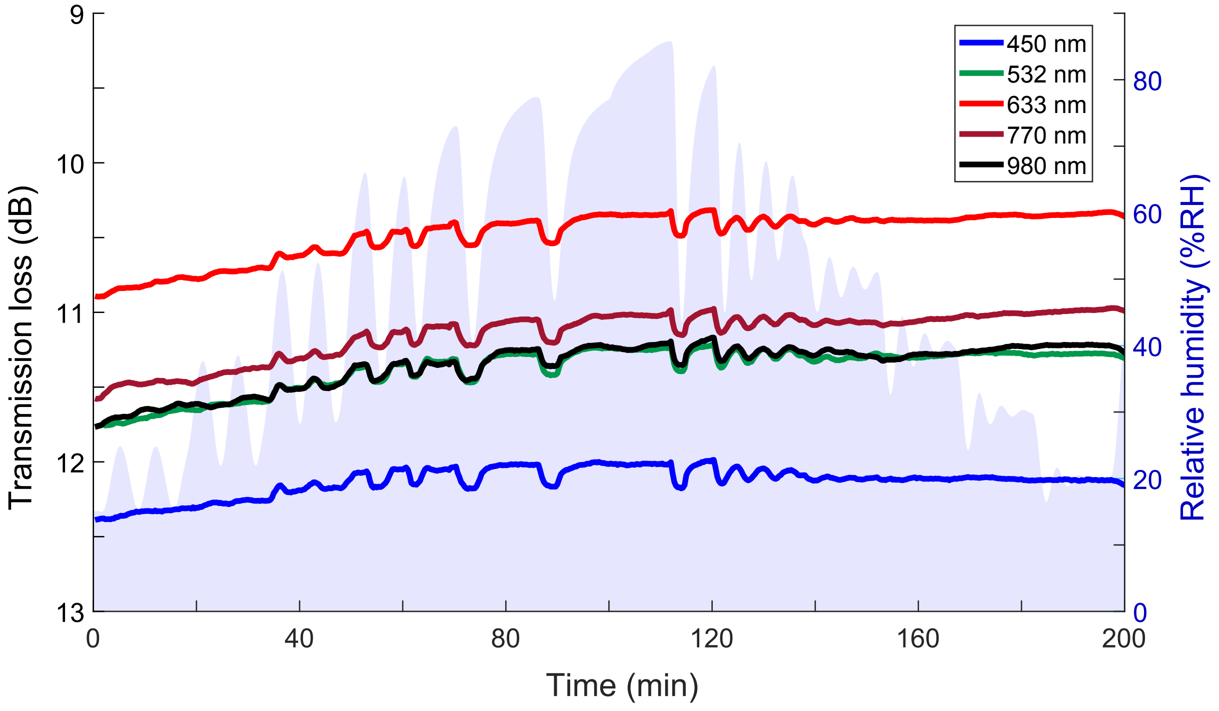

Figure S1. PMMA humidity sensitivity. Ramping humidity from 20 to 80 %RH yields virtually no changes in transmission. Rapid changes in humidity are seen as fringes when the condensed water layer on the fiber surface suddenly evaporates. Sample is a 5 cm piece of 1 mm thick PMMA MM POF (Farnell FDPF 4001 EH)

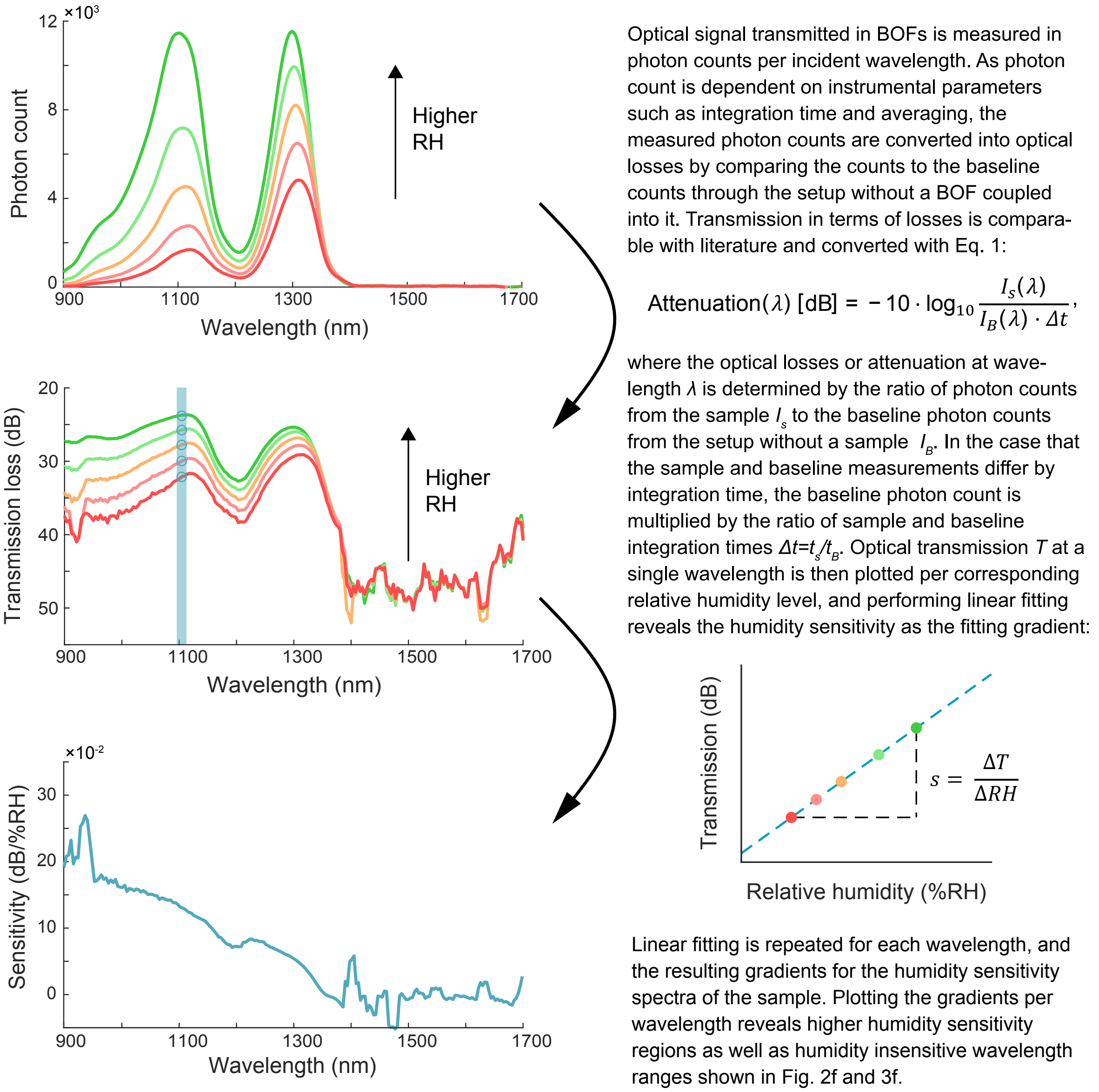

Figure S2. Converting photon count spectra into BOF humidity sensitivity spectra. A chart illustrating how photon counts measured with the spectrometers are first converted into transmission loss spectra in decibels and then compared to humidity values per each spectrum to evaluate BOF humidity sensitivity.

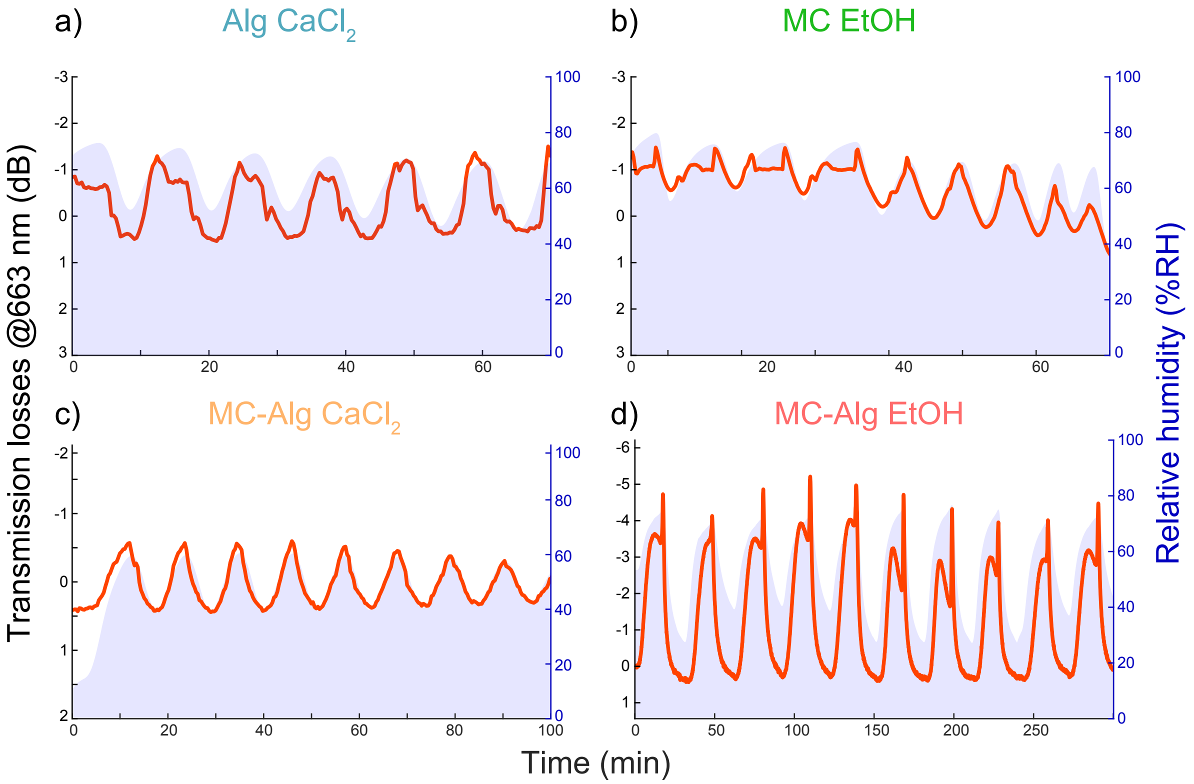

Figure S3. BOF humidity response time. Rapid changes in ambient humidity in single component BOFs a) Alg and b) MC as well as composite BOFs c) ionically coagulated MC-Alg and d) MC-Alg coagulated in ethanol were monitored over 70 – 280 min periods in the custom humidity chamber with 15 – 35 %RH cyclic variation. Normalized transmission changes in response to humidity variation at 633 nm reveal a good response in comparison to the commercial Linkam sensor.

Response time is estimated in relation to the reference RH sensor. As the humidity conditions were controlled using the Linkam RH95 Humidity Controller and the feedback loop with its RH sensor, we assume the Linkam sensor reading matches the chamber conditions, and thus the BOF sensor response time was estimated simply by comparing the time delay between the Linkam humidity reading and the BOF transmission changes. The time between the humidity controller

switching from decreasing to increasing humidity and the corresponding change in BOF transmission is illustrated in a-d. Response time estimation resolution was limited to 5 s due to instrument limitations as the Linkam sensor provided readings only every 5 s during an overnight measurement.

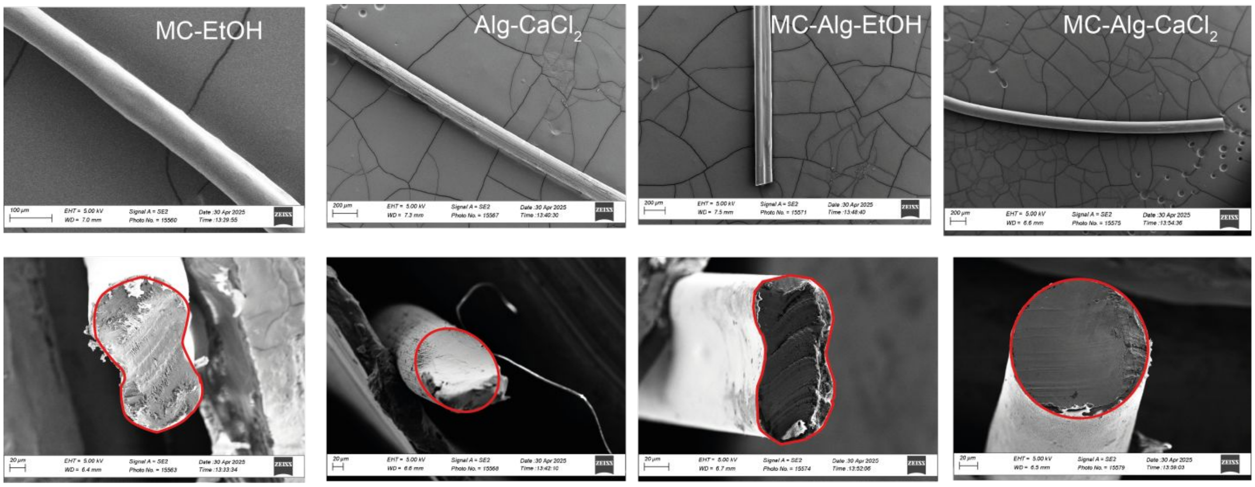

Figure S4. Scanning electron microscopy images of BOFs showing surface morphology (top) and corresponding cross-sectional views (bottom).

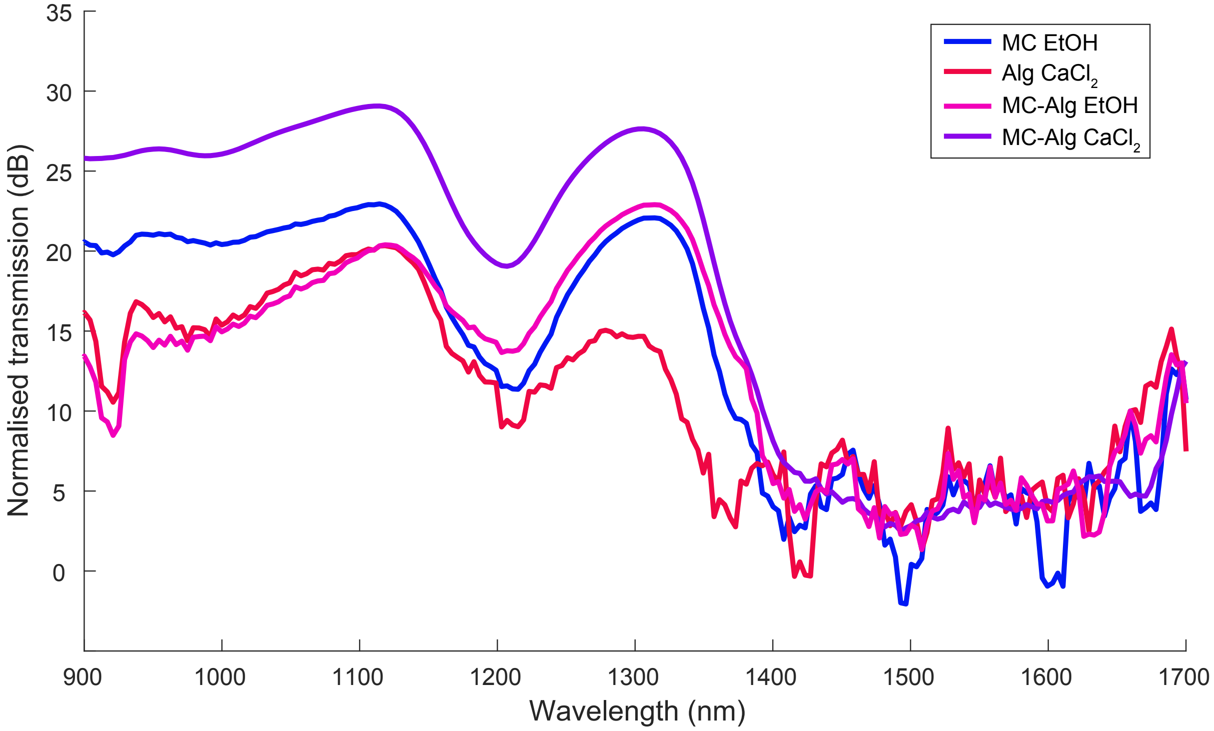

Figure S5. Normalized NIR spectra of BOFs. Transmission spectra from MC, Alg, and MC-Alg BOFs normalized by their negligible transmission between 1400 – 1600 nm. While ethanol coagulated MC and MC-Alg fibers show similar transmission around 1300 nm, single component MC fibers are better at transmitting wavelengths below 1150 nm. Similarly, ionically coagulated Alg and MC-Alg fibers both show higher relative transmission around 1100 nm than 1300 nm, albeit the transmission contrast is more pronounced with single component Alg fibers. Overall transmission is highest with ionically coagulated MC-Alg, combining the transparent MC material with the highly circular cross-section produced through ionic coagulation.

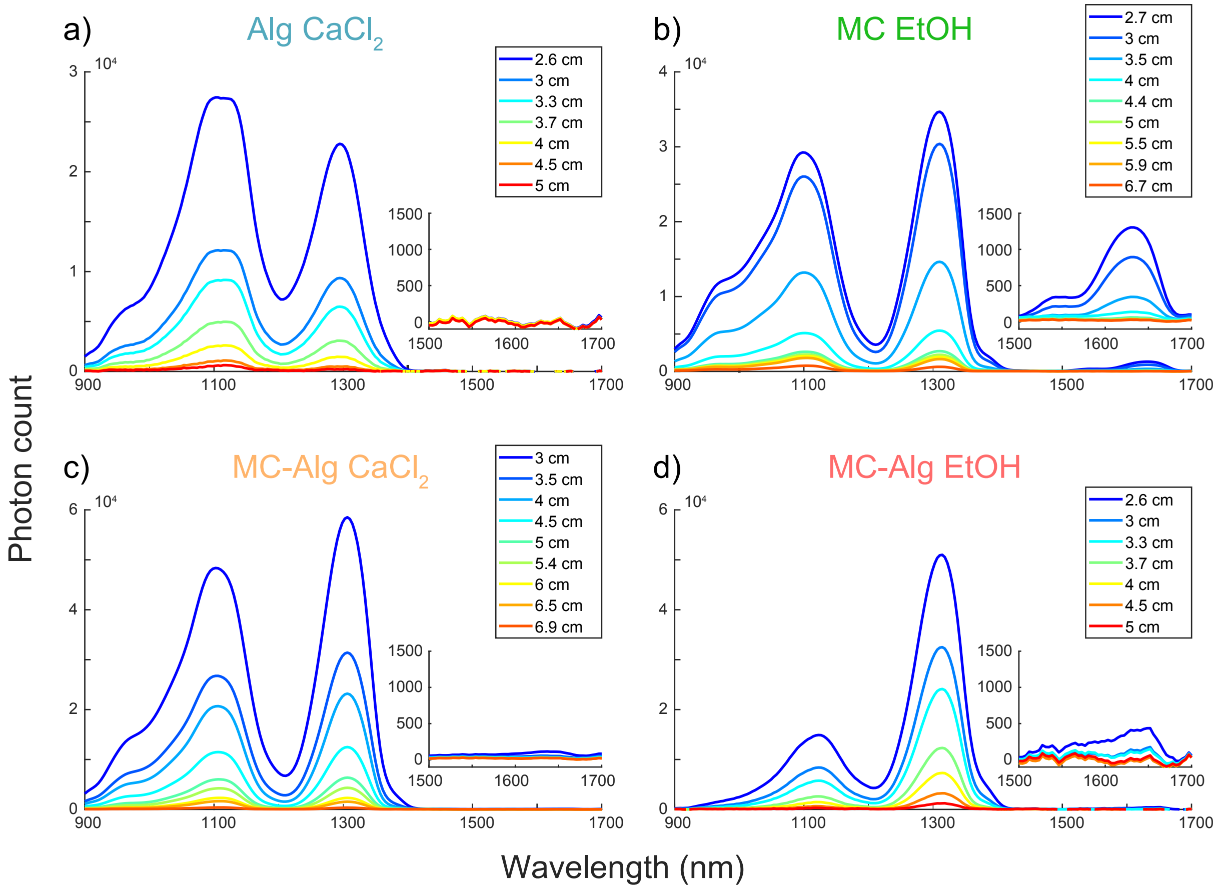

Figure S6. BOF transmission spectra. Photon count transmission spectra from single component BOFs a) Alg and b) MC as well as composite BOFs c) ionically coagulated MC-Alg and d) MC-Alg coagulated in ethanol. While the main text figures 2 and 3 present logarithmic BOF transmission spectra, these linear spectra highlight how ionically coagulated BOFs have no transmission above 1400 nm, while MC-containing fibers show limited transmission above 1600 nm. Ethanol-coagulated fibers also exhibit NIR transmission maxima around 1300 nm, while ionically coagulated fibers have similar or higher transmission peak around 1100 nm.

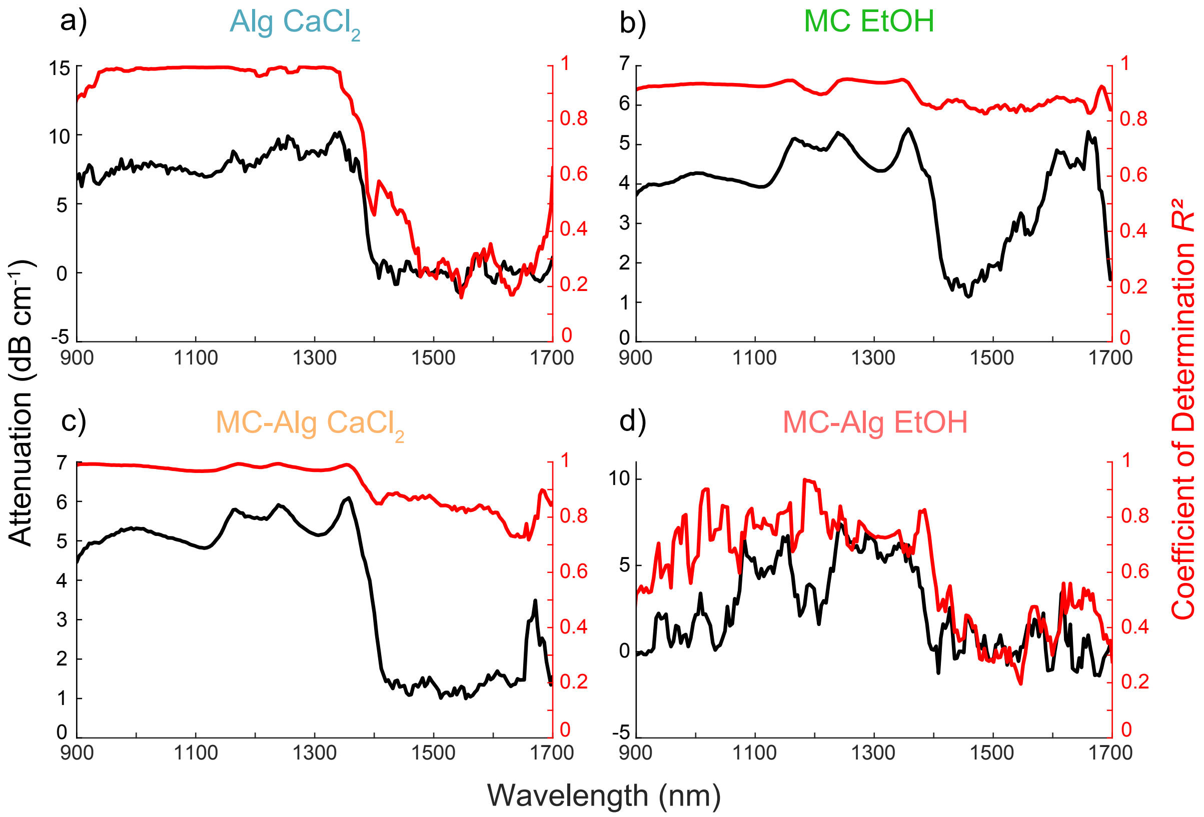

Figure S7. BOF NIR attenuation spectra. NIR attenuation spectra between 900 – 1700 nm derived by applying the Beer-Lambert law from Fig. S5 spectral data for single component BOFs a) Alg and b) MC as well as composite BOFs c) ionically coagulated MC-Alg and d) MC-Alg coagulated in ethanol. Attenuation spectra are presented with the black trace, while the coefficient of linear fitting correlation R^2 is presented with the red trace. While single component MC fibers show decent linear correlation across the spectrum, attenuation values above 1350 nm are unreliable for Alg and MC-Alg in EtOH. Correlation is also limited for MC-Alg in $CaCl_2$ above 1350 nm as virtually no transmission is observed in Fig. S5 (c).

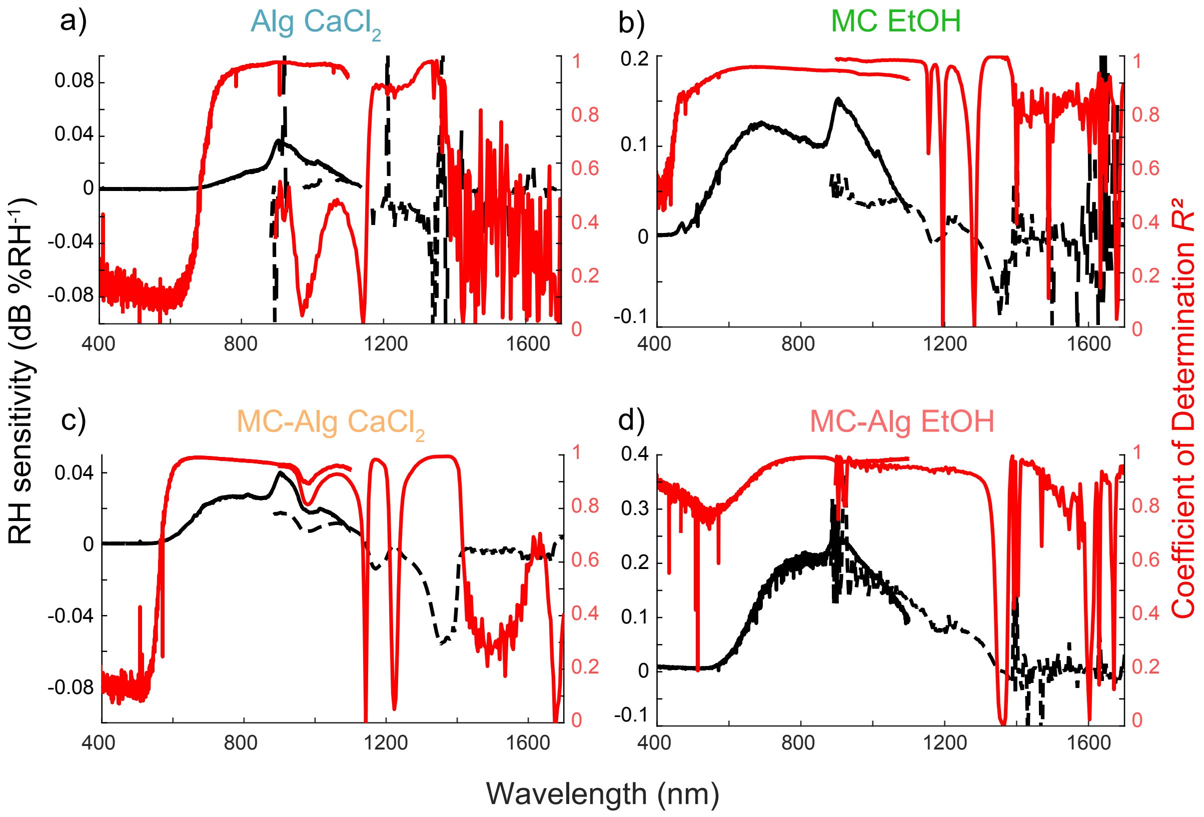

Figure S8. BOF humidity sensitivity spectra. Raw humidity sensitivity spectra without applied smoothing in UV-Vis (solid line) and NIR (dashed line) determined through the procedure described in Fig. S2 for single component BOFs a) Alg and b) MC as well as composite BOFs c) ionically coagulated MC-Alg and d) MC-Alg coagulated in ethanol. Humidity sensitivity spectra are presented with the black trace, while the coefficient of linear fitting correlation R^2 is presented with the red trace. While one-component MC fiber displays mostly reliable humidity sensitivity values ($R^2 > 0.9$) in Vis-NIR between 500 – 1400 nm, one-component Alg fiber sensitivity linearity only applies between 800 – 1400 nm. Similarly, both ionically and ethanol-coagulated MC-Alg fibers show mostly good coefficient of correlation in humidity sensitivity between 600 – 1400 nm. All BOFs show poor coefficients of determination around hydroxyl absorption peaks:

1143 and 1223 nm for ionically coagulated MC-Alg and 1360 nm for ethanol-coagulated MC-Alg, for example.

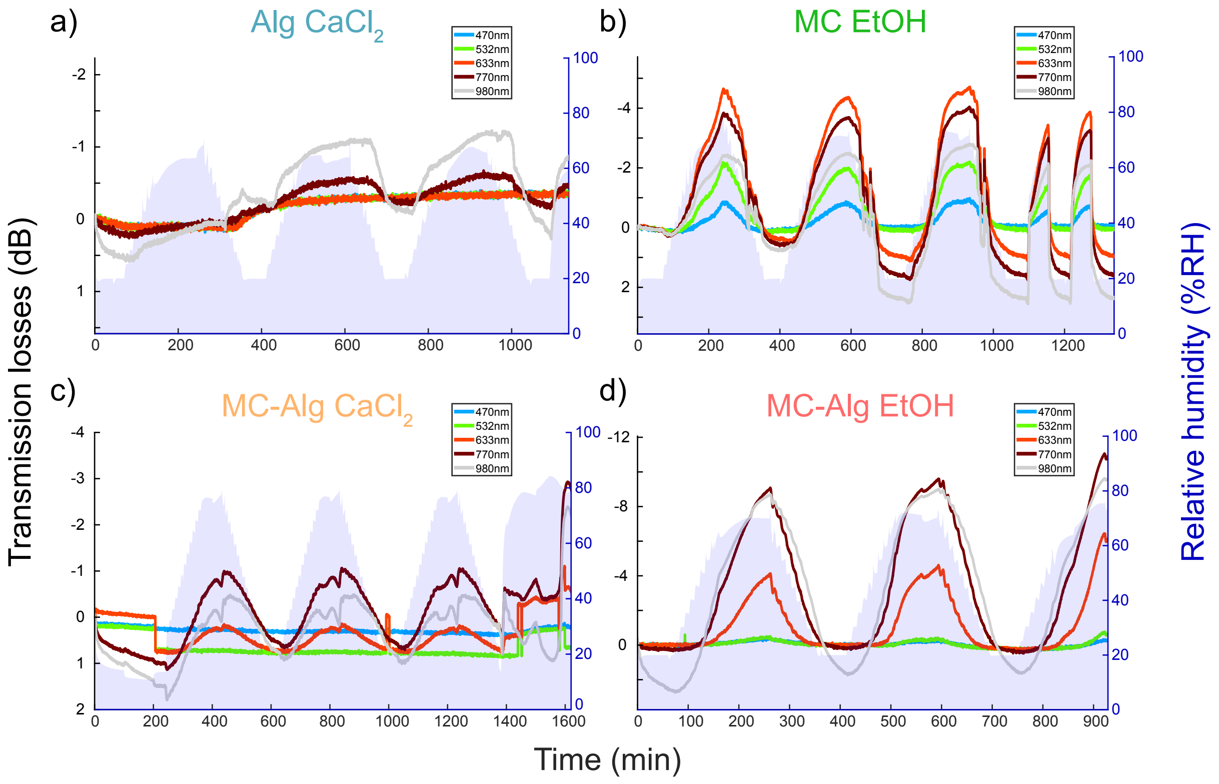

Figure S9. BOF Vis transmission during RH ramping. Normalized transmission at wavelengths of 470, 532, 633, 770, and 980 nm through single component BOFs a) Alg and b) MC as well as composite BOFs c) ionically coagulated MC-Alg and d) MC-Alg coagulated in ethanol are presented with RH ramping values. Transmission at different wavelengths (colored traces) varies as a function of ambient RH (blue area on the background). Ionically coagulated Alg and MC-Alg fibers produce the weakest and least reproducible RH response, while MC and MC-Alg fibers coagulated in ethanol display higher sensitivity and more reproducible response.

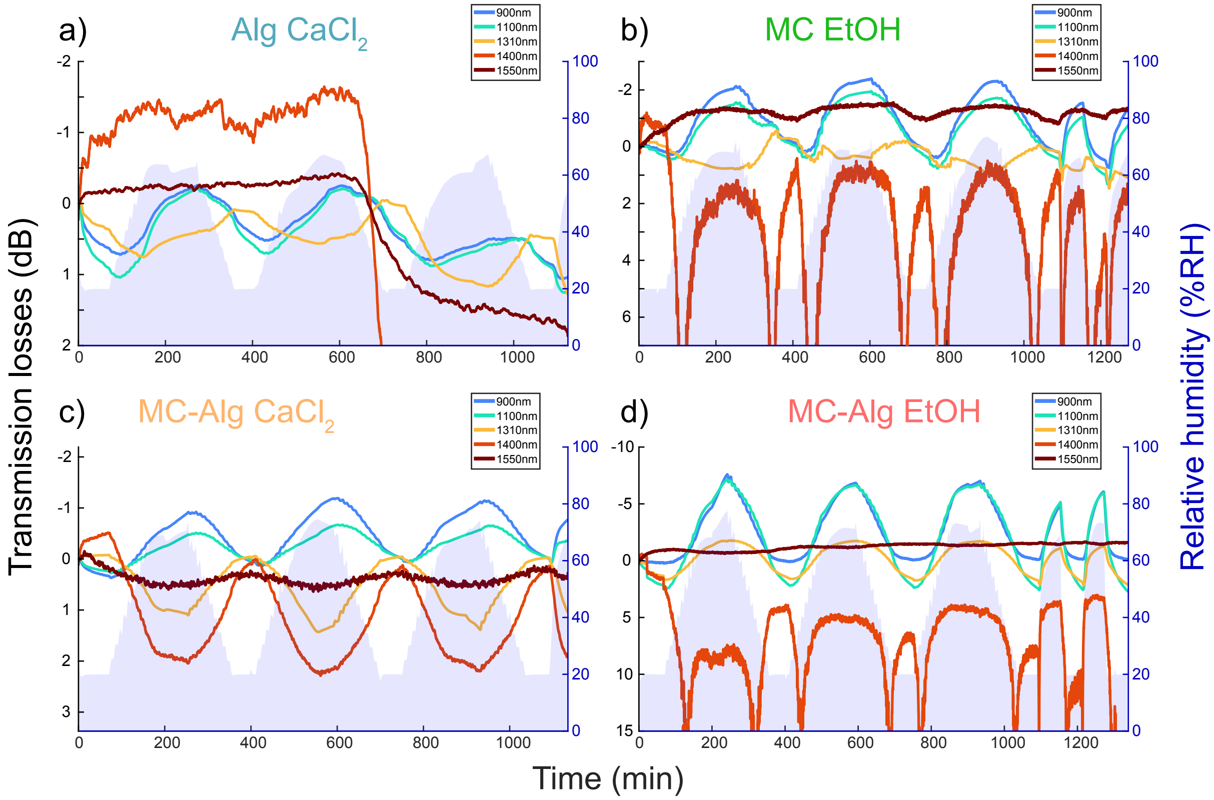

Figure S10. BOF NIR transmission during RH ramping. Transmission at select wavelengths of 900, 980, 1100, 1200, 1310, 1400, and 1550 nm through single component BOFs a) Alg and b) MC as well as composite BOFs c) ionically coagulated MC-Alg and d) MC-Alg coagulated in ethanol are presented with RH ramping values. Hydroxyl absorption around 1400 nm is seen in most samples as significant transmission losses at higher RH, while wavelengths above 1200 nm exhibit increased transmission at higher RH. MC-Alg in EtOH shows the highest RH sensitivity with good repeatability in humidity response, note the larger y-axis with MC-Alg EtOH.

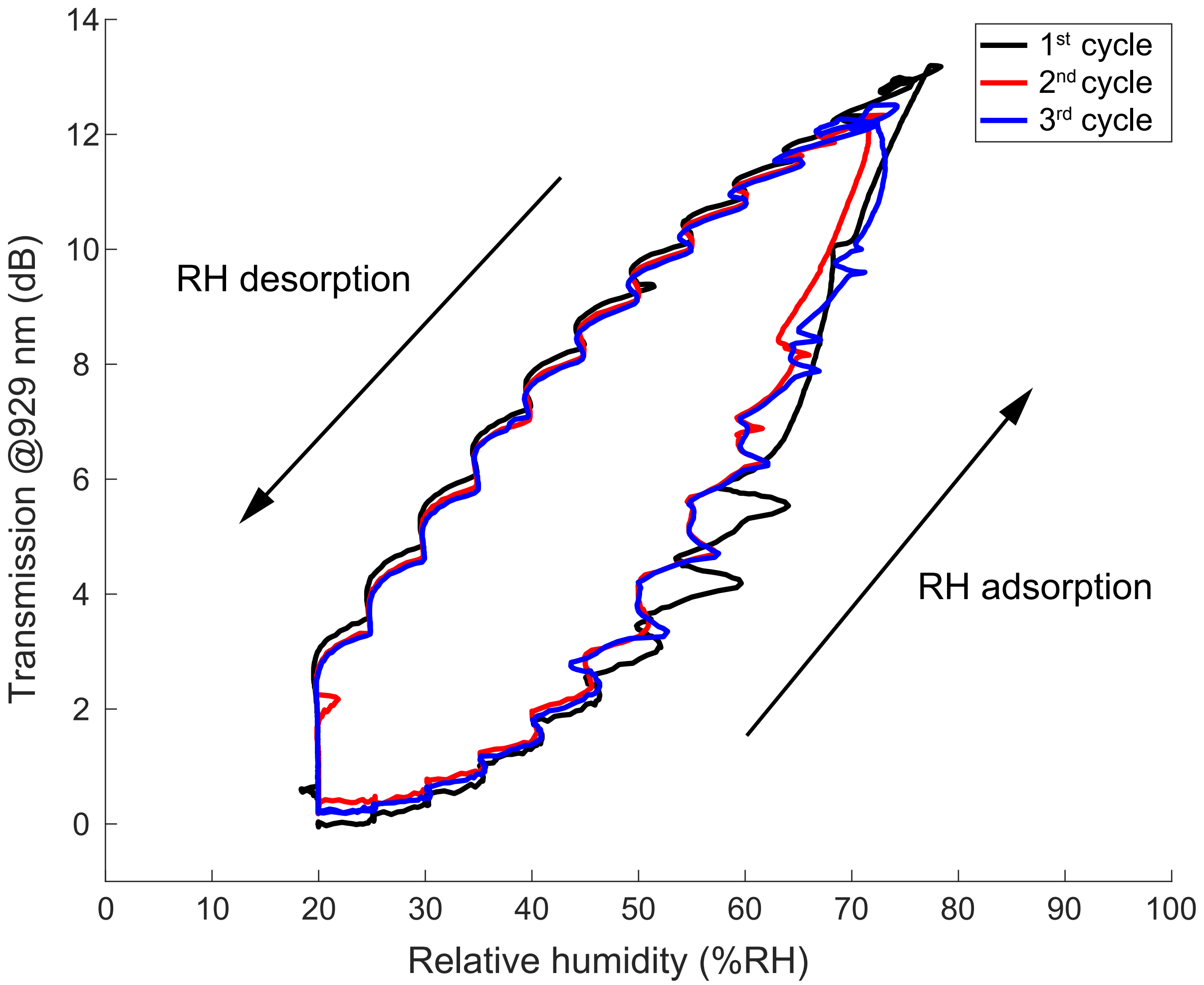

Figure S11. MC-Alg EtOH RH hysteresis. Normalized transmission changes at 929 nm in repeated cycles of increasing and decreasing RH reveals hysteresis in BOFs. The presented example from MC-Alg composite fiber coagulated in ethanol shows that after initial first-cycle relaxation, second and third cycle humidity sensitivity is repeatable with hysteresis up to 6 dB or 11.1 %. The lowest maximum RH hysteresis measured from the BOFs was found to be 2.28 % in MC-Alg $CaCl_2$ fibers.

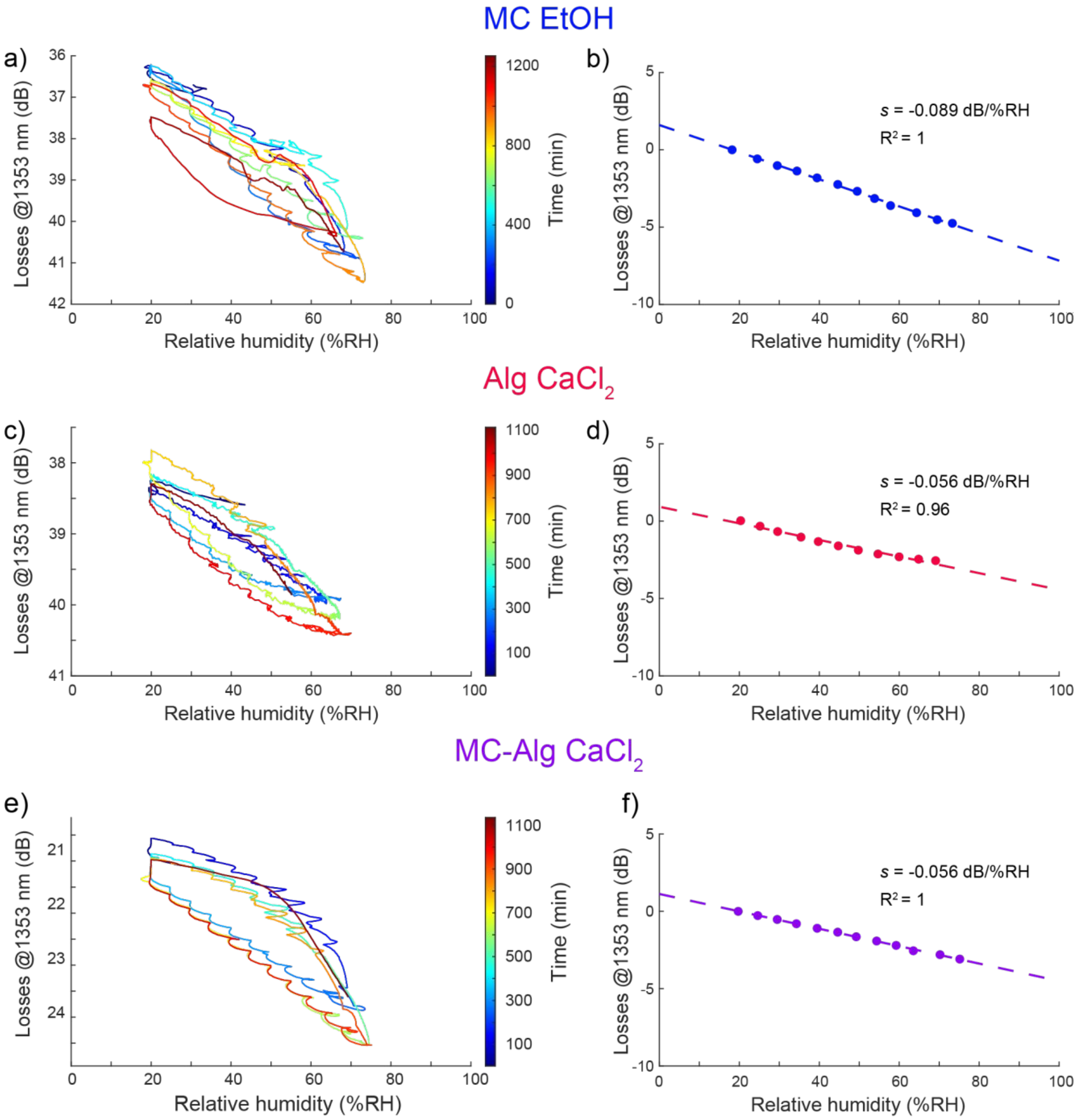

Figure S12. Humidity sensitivity at 1353 nm of: a & b) Ethanol coagulated MC BOFs, c & d) Ionically coagulated Alg BOFs, and e & f) Ionically coagulated MC-Alg composite BOFs. Transmission changes close to the hydroxyl ion absorption band at 1353 nm in repeated cycles of increasing and decreasing RH reveals lower hysteresis (2–3 %) in BOFs than at peak sensitivity wavelengths around 900 nm. MC, Alg, and MC-Alg coagulated in $CaCl_2$ all show negative linear humidity sensitivity where transmission decreases as ambient humidity increases, suggesting absorption losses from increased water intake.

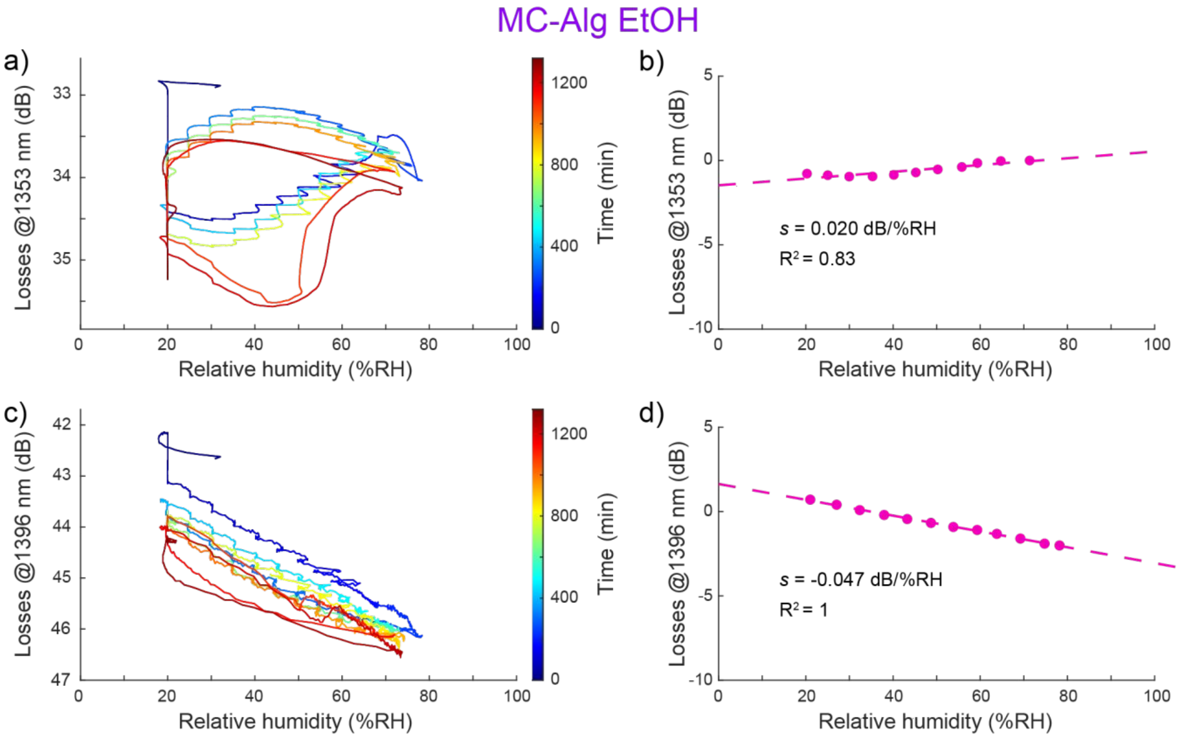

Figure S13. MC-Alg EtOH humidity sensitivity at 1353 nm and 1396 nm. a & b) Transmission changes close to the hydroxyl ion absorption band at 1353 nm in repeated cycles of increasing and decreasing RH do not correspond to the behavior observed in other BOF samples. MC-Alg coagulated in ethanol shows aberrant RH response with poor linearity and possibly slightly positive humidity sensitivity. c & d) At another wavelength of 1396 nm also close to the hydroxyl ion absorption band, similar negative linear humidity sensitivity is observed as with other BOF samples.

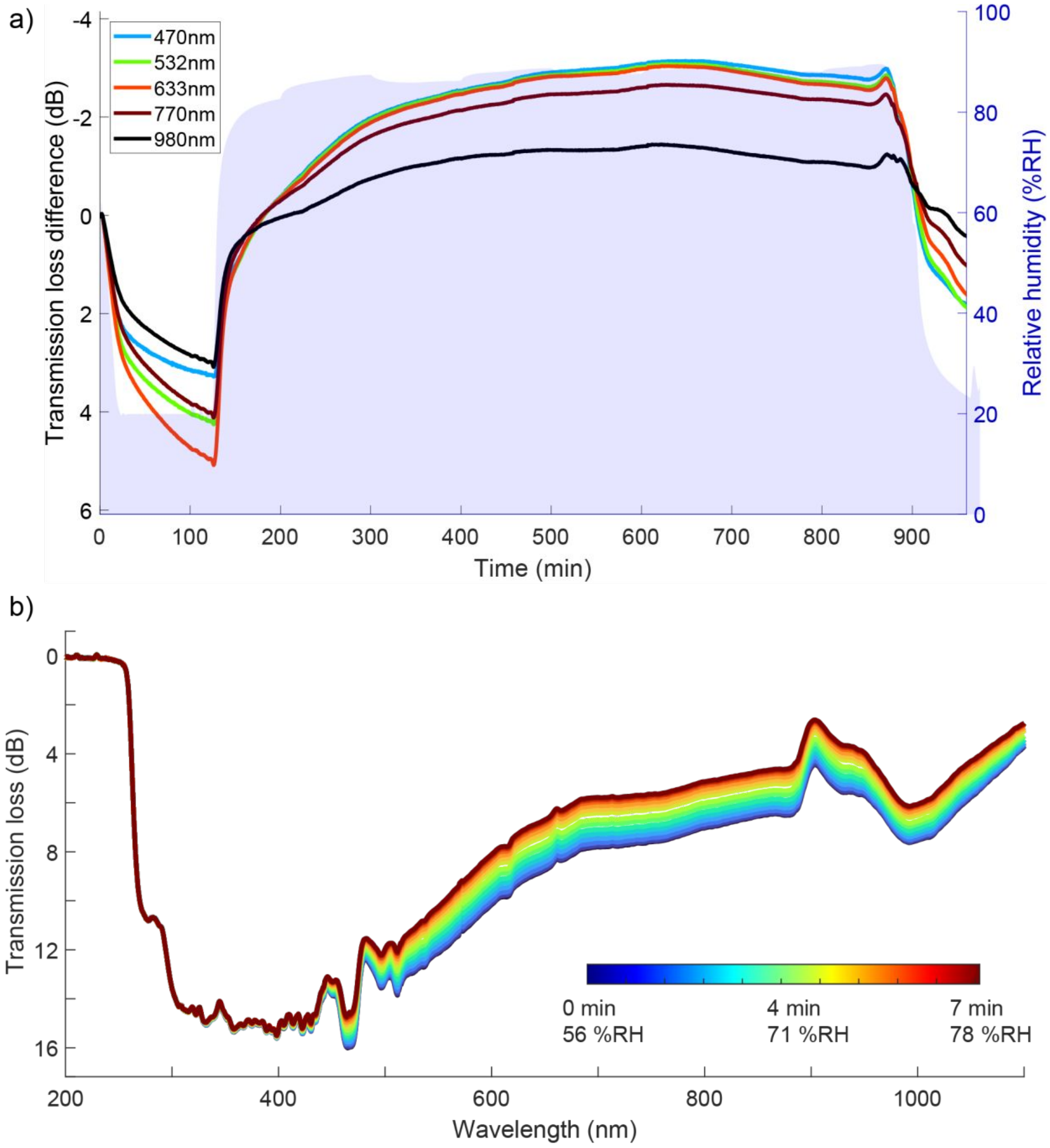

Figure S14. MC BOF in high humidity. a) Single component MC fiber exposed to consecutive 2 h drying and 12 h wetting steps in the custom humidity experiment chamber at 20 %RH and 85 %RH, respectively. As water desorbs from MC BOF during the drying step, optical losses increase, while optical transmission improves during the longer wetting step. The 2 h drying step was not long enough for water desorption to reach equilibrium, but changes in transmission due to moisture absorption saturated after 6 h exposure to 85 %RH. b) Rapid increase in RH from 56 to 78 %RH over 7 min produces equally rapid 2-3 dB improvements in transmission across the 450-1100 nm transmission window for single component MC fibers.

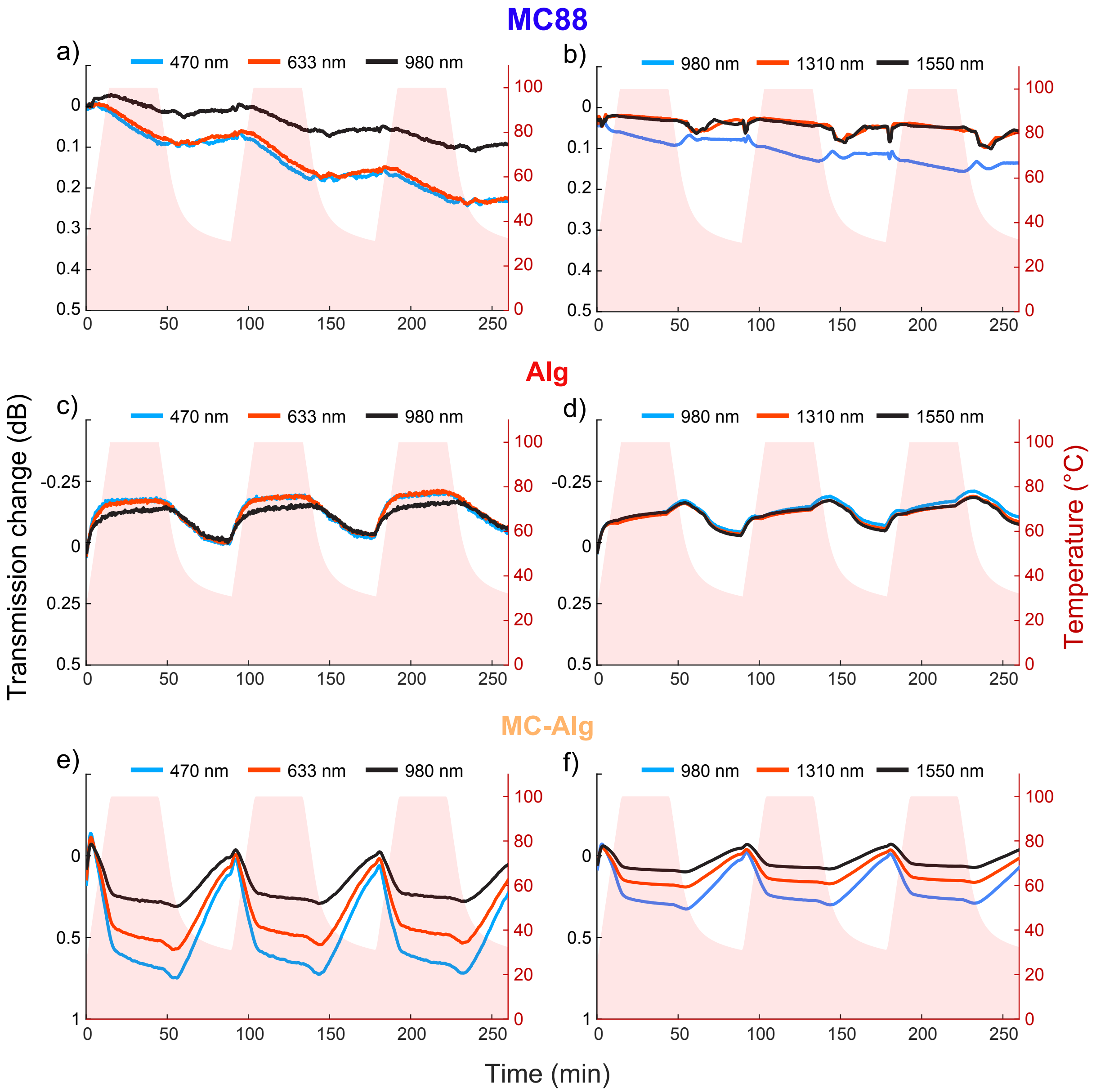

Figure S15. Biopolymer thin film temperature sensitivity. Normalized transmission at wavelengths of 470, 633, 980, 1310, and 1550 nm through MC, Alg, and MC-Alg biopolymer films during repeated heating-cooling cycles from 25 to 100 ˚C. MC films display minimal and, in this timescale, nonreversible transmission decreases upon heating to 100 ˚C in both a) UV-Vis and b) NIR spectra. 0.1 – 0.2 dB decrease in MC film transmission presumably due to humidity

evaporation. Curiously, single component Alg films show low but and reversible 0.2 dB increase in transmission upon heating in both c) UV-Vis and d) NIR, suggesting a temperature sensitivity albeit an order of magnitude weaker than its humidity response. Composite MC-Alg film shows the greatest reversible temperature sensitivity of 0.1 – 0.7 dB changes across the 25 to 100 ˚C range with e) UV-Vis sensitivity particularly at lower Vis wavelengths being considerably more sensitive to temperature changes than at f) NIR spectra, where transmission changes were more minute at longer wavelengths.

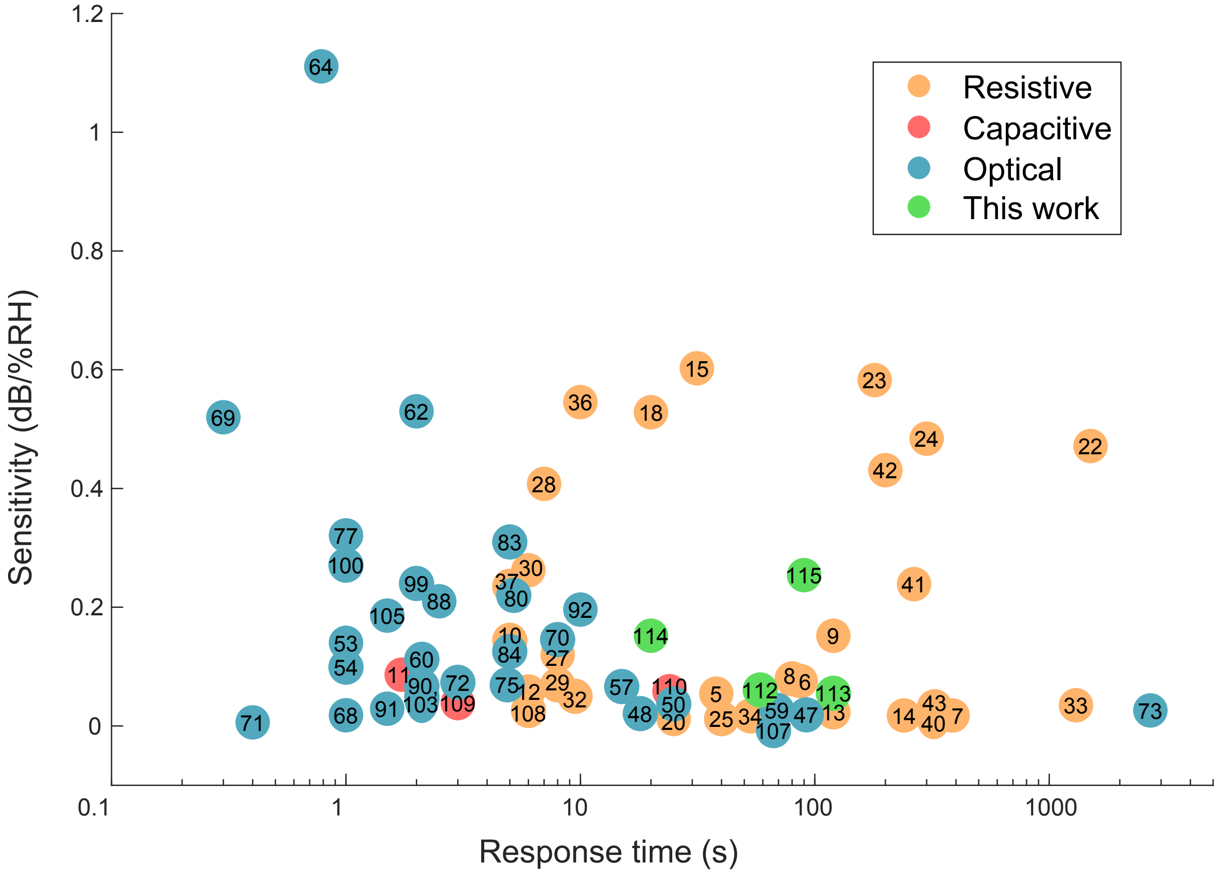

Figure S16. Comparison of humidity sensors. Comparable literature values for humidity sensitivity in resistive, capacitive, and optical sensor systems by device biopolymer content published between 1988 – 2025 with both reported sensitivity and response time. Best-performing BOF

reported in this work shows above average RH sensitivity compared to other optical sensing systems. Literature references listed with their corresponding index in Table S1.

Table S1. List of literature references used for comparative study

#	Short reference	DOI
1	Anisimov et al. 2022 ACS AMI	https://doi.org/10.1021/acsapm.2c01078
2	Anisimov et al. 2022 ACS AMI	https://doi.org/10.1021/acsapm.2c01078
3	Anisimov et al. 2022 ACS AMI	https://doi.org/10.1021/acsapm.2c01078
4	Ramaprasad et al. 2010 Sens. Act. B	https://doi.org/10.1016/j.snb.2010.05.044
5	Zhou et al. 2015 Polymer Bulletin	https://doi.org/10.1007/s00289-015-1509-y
6	Patil et al. 2010 Sens. Act. B	https://doi.org/10.1016/j.snb.2010.04.046
7	Nagaraju et al. 2014 J. Eng.	https://doi.org/10.1155/2014/925020
8	Kotresh et al. 2015 Adv.Mater. Lett.	https://doi.org/10.5185/amlett.2015.5795
9	Kuang et al. 2007 JACS	https://doi.org/10.1021/ja070788m
10	Guo et al. 2017 Nanoscale	https://doi.org/10.1039/C7NR01016H
11	Awais et al. 2020 Sci. Rep.	https://doi.org/10.1038/s41598-020-62397-x
12	Qian et al. 2016 Sci. Rep.	https://doi.org/10.1038/srep25574
13	Farooq et al. 2019 J. Electron. Mater.	https://doi.org/10.1007/s11664-019-06931-2
14	Pawar et al. 2015 RSC Adv.	https://doi.org/10.1039/C5RA17253E
15	Kano et al. 2018 ACS Sus. Chem. Eng.	https://doi.org/10.1021/acssuschemeng.8b02550
16	Kanaparthi et al. 2017 Electroanalysis	https://doi.org/10.1002/elan.201700438
17	Mahadeva et al. 2011 Sens. Actuators, A	https://doi.org/10.1016/j.sna.2010.10.018
18	Barmpakos et al. 2017 J. -Phys. Conf. Ser.	https://doi.org/10.1088/1742-6596/931/1/012003
19	Kano et al. 2017 ACS Sensors	http://dx.doi.org/10.1021/acssensors.7b00199
20	Shukla et al. 2013 Int. J. Bio. Macromolecules	https://doi.org/10.1016/j.ijbiomac.2013.10.014
21	Qi et al. 2013 J. Mater. Chem. A	https://doi.org/10.1039/c2ta00882c
22	Güder et al. 2016 Angew Chem	https://doi.org/10.1002/anie.201511805
23	Quddious et al. 2016 MDPI Sensors	https://doi.org/10.3390/s16122073
24	Zhao et al. 2017 ACS AMI	https://doi.org/10.1021/acsami.7b05181
25	Sahatiya et al. 2018 ACS AMI	http://dx.doi.org/10.1021/acsami.8b00245
26	Chen et al. 2018 J. Mat. Chem. A	https://doi.org/10.1039/C8TA00618K
27	Zhang et al. 2019 J. Mat. Chem. A	https://doi.org/10.1039/C9TC01254K
28	Zhao et al. 2019 IEEE Sens. J.	https://doi.org/10.1109/JSEN.2018.2879826
29	Sahoo et al. 2020 Mat. Sci. Semicon. Processing	https://doi.org/10.1016/j.mssp.2019.104699
30	Wang et al. 2020 ACS AMI	https://dx.doi.org/10.1021/acsami.9b22754
31	Kalim et al. 2020 Ceramics Int.	https://doi.org/10.1016/j.ceramint.2020.07.031
32	Khalifa et al. 2020 Synthetic Metals	https://doi.org/10.1016/j.synthmet.2020.116420
33	Sandhu et al. 2020 IEEE Sens. J.	https://doi.org/10.1109/JSEN.2020.3001599
34	Bhattacharjee et al. 2019 Sensors and Actuators A	https://doi.org/10.1016/j.sna.2018.11.034
35	Yuan et al. 2016 RSC Adv.	https://doi.org/10.1039/C6RA03050E
36	Kotresh et al. 2016 Cellulose	https://doi.org/10.1007/s10570-016-1035-6
37	Barras et al. 2017 Flex. Print. Electron.	https://doi.org/10.1088/2058-8585/aa5ef9
38	Hashim et al. 2018 J. Mater. Sci. Mater. Electron.	https://doi.org/10.1007/s10854-018-9257-z
39	Xu et al. 2016 Comp. Sci. Tech.	https://doi.org/10.1016/j.compscitech.2016.05.014
40	Zhu et al. 2019 Langmuir	https://doi.org/10.1021/acs.langmuir.8b04259
41	Syrový et al. 2019 J. Appl. Polym. Sci.	https://doi.org/10.1002/app.47920
42	Syrový et al. 2019 J. Appl. Polym. Sci.	https://doi.org/10.1002/app.47920

43	Zhu et al. 2021 Chem. Eng. J.	https://doi.org/10.1016/j.cej.2020.127105
44	Verma et al. 2014 Optics & Laser Tech.	https://doi.org/10.1016/j.optlastec.2013.10.007
45	Yadav et al. 2013 Optics & Laser Tech.	http://dx.doi.org/10.1016/j.optlastec.2012.12.011
46	Yadav et al. 2013 Optics & Laser Tech.	http://dx.doi.org/10.1016/j.optlastec.2012.12.011
47	Dixit et al. 2007 Opt. Rev.	https://doi.org/10.1007/s10043-007-0186-y
48	Yadav et al. 2010 Sens. Actuators B: Chem.	https://doi.org/10.1016/j.snb.2010.05.046
49	Singh et al. 2012 Appl. Surf. Sci.	https://doi.org/10.1016/j.apsusc.2012.05.091
50	Yadav et al. 2012 Optics & Laser Tech.	https://doi.org/10.1016/j.optlastec.2011.12.041
51	Shukla et al. 2004 Sens. Actuators B: Chem.	https://doi.org/10.1016/j.snb.2003.05.001
52	Tang et al. 2018 Appl. Opt.	https://doi.org/10.1364/AO.57.002539
53	Wang et al. 2017 J. Light. Technol.	https://doi.org/10.1109/JLT.2017.2730484
54	Matthew et al. 2013 IEEE Sens. J.	https://doi.org/10.1109/JSEN.2013.2238229
55	Liu et al. 2012 Opt. Express	https://doi.org/10.1364/OE.20.019404
56	Aneesh et al. 2012 Appl. Opt.	https://doi.org/10.1364/AO.51.002164
57	Mitschke et al. 1989 Opt. Lett.	https://doi.org/10.1364/OL.14.000967
58	Brook et al. 1997 Sens. Actuators B Chem	https://doi.org/10.1016/S0925-4005(97)80217-1
59	Otsuki et al. 1998 Sens. Actuators B Chem	https://doi.org/10.1016/S0925-4005(98)00296-2
60	Muto et al. 2003 Meas. Sci. Technol.	https://doi.org/10.1088/0957-0233/14/6/306
61	Gaston et al. 2003 IEEE Sens. J.	https://doi.org/10.1109/JSEN.2003.820349
62	Gaston et al. 2004 Appl. Opt.	https://doi.org/10.1364/AO.43.004127
63	Tan et al. 2005 Sens. Actuators B Chem	https://doi.org/10.1016/j.snb.2005.02.012
64	Mathew et al. 2011 Opt. Laser Technol.	https://doi.org/10.1016/j.optlastec.2011.03.028
65	Li et al. 2013 IEEE Sens. J.	https://doi.org/10.1109/JSEN.2012.2234100
66	Chen et al. 2014 IEEE Electron Device Lett.	https://doi.org/10.1109/LED.2014.2310741
67	Alvarez-Herrero et al. 2004 IEEE Sens. J.	https://doi.org/10.1109/JSEN.2003.822214
68	Khijwania et al. 2005 Sens. Actuators B Chem	https://doi.org/10.1016/j.snb.2004.05.012
69	Corres et al. 2007 Sens. Actuators B Chem	https://doi.org/10.1016/j.snb.2006.06.008
70	Vijayan et al. 2008 Sens. Actuators B Chem	https://doi.org/10.1016/j.snb.2007.07.113
71	Akita et al. 2010 Sens. Actuators B Chem	https://doi.org/10.1016/j.snb.2010.03.049
72	Tay et al. 2004 Microw. Opt. Technol. Lett.	https://doi.org/10.1002/mop.20479
73	Yeo et al. 2005 Sens. Actuators B Chem	https://doi.org/10.1016/j.snb.2005.01.033
74	Liu et al. 2007 IEEE Photon. Technol. Lett.	https://doi.org/10.1109/LPT.2007.897551
75	Huang et al. 2007 Sens. Actuators B Chem	https://doi.org/10.1016/j.snb.2007.05.007
76	Viegas et al. 2009 Meas. Sci. Technol.	http://dx.doi.org/10.1088/0957-0233/20/3/034002
77	Ouyang et al. 2017 Opt. Express	https://doi.org/10.1364/OE.25.009823
78	Rahman et al. 2015 IOP Conference: Mat. Sci.Eng.	https://doi.org/10.1088/1757-899X/99/1/012025
79	Sun et al. 2016 Sens. Actuators B Chem.	https://doi.org/10.1016/j.snb.2016.03.102
80	Gao et al. 2016 Sens. Actuators B Chem.	https://doi.org/10.1016/j.snb.2015.08.108
81	Kim et al. 2010 Sens. Actuators A Phys.	https://doi.org/10.1016/j.sna.2010.01.032
82	Gaston et al. 2004 Appl. Opt.	https://doi.org/10.1364/AO.43.004127
83	Xiao et al. 2014 Opt. Express	https://doi.org/10.1364/OE.22.031555
84	Bariain et al. 2000 Sens. Actuators B Chem.	https://doi.org/10.1016/S0925-4005(00)00524-4
85	Bariain et al. 2001 Sens. Actuators B Chem.	https://doi.org/10.1016/S0925-4005(01)00899-1
86	Xuan et al. 2014 Sci. Rep.	https://doi.org/10.1038/srep07206
87	Mallik et al. 2016 Opt. Express	https://doi.org/10.1364/OE.24.021216
88	Luo et al. 2018 IEEE Sens. J.	https://doi.org/10.1109/JSEN.2018.2865035
89	Wang et al. 2016 Sens. Actuators B Chem.	https://doi.org/10.1016/j.snb.2016.05.020
90	Arregui et al. 2000 IEICE Trans. Electr.	No DOI available, MLA citation: ARREGUI, Francisco J., et al. "Optical fiber humidity sensor with a fast response time using the ionic self-assembly method." IEICE Transactions on electronics 83.3 (2000): 360-365.
91	Arregui et al. 2002 IEEE Sens. J.	https://doi.org/10.1109/JSEN.2002.804577
92	Xia et al. 2013 Sens. Actuators A Phys.	https://doi.org/10.1016/j.sna.2012.10.041

93	Corres et al. 2006 IEEE Photon. Technol. Lett.	https://doi.org/10.1109/LPT.2006.873568
94	Liu et al. 2015 IEEE Sens. J.	https://doi.org/10.1109/JSEN.2015.2389519
95	Chen et al. 2012 IEEE Journal of Selected Topics in Quantum Electronics	https://doi.org/10.1109/JSTQE.2012.2194729
96	Corres et al. 2006 J. Lightwave Technol.	No DOI available, MLA citation: Corres, Jesús M., Francisco J. Arregui, and Ignacio R. Matias. "Design of humidity sensors based on tapered optical fibers." Journal of Lightwave Technology 24.11 (2006): 4329-4336.
97	Zhou et al. 1988 Anal. Chem.	https://doi.org/10.1021/ac00171a035
98	Tao et al. 2004 IEEE Sens. J.	https://doi.org/10.1109/JSEN.2004.827274
99	Jindal et al. 2001 Opt. Eng.	https://doi.org/10.1117/1.1465429
100	Khijwania et al. 2005 Opt. Eng.	https://doi.org/10.1117/1.1870753
101	Giaccari et al. 2001 Optica BGPP	https://doi.org/10.1364/BGPP.2001.BFB2
102	Kronenberg et al. 2002 Opt. Lett.	https://doi.org/10.1364/OL.27.001385
103	Li et al. 2012 IEEE Sens. J.	https://doi.org/10.1109/JSEN.2011.2181358
104	Shao et al. 2014 Optics and Lasers in Engineering	https://doi.org/10.1016/j.optlaseng.2013.07.023
105	Ascorbe et al. 2016 Sens. Actuators B Chem.	https://doi.org/10.1016/j.snb.2016.04.045
106	Ni et al. 2017 Optical Fiber Technology	https://doi.org/10.1016/j.yofte.2016.11.005
107	Hartings et al. 2018 Sens. Actuators B Chem.	https://doi.org/10.1016/j.snb.2018.03.065
108	Han et al. 2012 J. Phys. Chem. C	https://doi.org/10.1021/jp3080223
109	Ducéré et al. 2005 Sens. Actuators B Chem.	https://doi.org/10.1016/j.snb.2004.08.028
110	Molina-Lopez et al. 2012 Sens. Actuators B Chem.	https://doi.org/10.1016/j.snb.2012.02.042
111	Zhao et al. 2019 ACS AMI	https://doi.org/10.1021/acsami.7b09184
112	**Alg $CaCl_2$**	**This work**
113	**MC-Alg $CaCl_2$**	**This work**
114	**MC EtOH**	**This work**
115	**MC-Alg EtOH**	**This work**

Table S1. Sensor comparison literature references. Literature values and the corresponding index (#) for main text Fig. 6 and Fig. S10.

REFERENCES

1. Kao, K. C.; Hockham, G. A. Dielectric-fibre surface waveguides for optical frequencies. *Proc. IEEE* **1966**, *113*, 1151–1158.
